# Mathematical models for the Notch and Wnt signaling pathways and the crosstalk between them during somitogenesis

**DOI:** 10.1186/1742-4682-10-27

**Published:** 2013-04-20

**Authors:** Hong-yan Wang, Yan-xin Huang, Yun-feng Qi, Yu Zhang, Yong-li Bao, Lu-guo Sun, Li-hua Zheng, Yu-wei Zhang, Zhi-qiang Ma, Yu-xin Li

**Affiliations:** 1 National Engineering Laboratory for Druggable Gene and Protein Screening, Northeast Normal University, Changchun 130024, China; 2 Research Center of Agriculture and Medicine Gene Engineering of Ministry of Education, Northeast Normal University, ChangChun 130024, China; 3 School of Computer Science and Information Technology, Northeast Normal University, Changchun 130117, China

## Abstract

**Background:**

Somitogenesis is a fundamental characteristic feature of development in various animal embryos. Molecular evidence has proved that the Notch and Wnt pathways play important roles in regulating the process of somitogenesis and there is crosstalk between these two pathways. However, it is difficult to investigate the detailed mechanism of these two pathways and their interactions in somitogenesis through biological experiments. In recent years some mathematical models have been proposed for the purpose of studying the dynamics of the Notch and Wnt pathways in somitogenesis. Unfortunately, only a few of these models have explored the interactions between them.

**Results:**

In this study, we have proposed three mathematical models for the Notch signalling pathway alone, the Wnt signalling pathway alone, and the interactions between them. These models can simulate the dynamics of the Notch and Wnt pathways in somitogenesis, and are capable of reproducing the observations derived from wet experiments. They were used to investigate the molecular mechanisms of the Notch and Wnt pathways and their crosstalk in somitogenesis through the model simulations.

**Conclusions:**

Three mathematical models are proposed for the Notch and Wnt pathways and their interaction during somitogenesis. The simulations demonstrate that the extracellular Notch and Wnt signals are essential for the oscillating expressions of both Notch and Wnt target genes. Moreover, the internal negative feedback loops and the three levels of crosstalk between these pathways play important but distinct roles in maintaining the system oscillation. In addition, the results of the parameter sensitivity analysis of the models indicate that the Notch pathway is more sensitive to perturbation in somitogenesis.

## Background

Animals have a segmented aspect of the body axis and somitogenesis has long been thought to be a key aspect of the basic design of animals. In the early developing animal embryo the body is organized in a series of embryonic tissue masses called somites [1]. Somites are progressively pinched off in pairs from the anterior end of two rods of mesenchymal tissue called the presomitic mesoderm (PSM) [2]. It is accepted that somite formation is controlled by a complicated gene network named the segmentation clock. For nearly a decade it has been known that the Notch pathway, the Wnt pathway and the fibroblast growth factor (FGF) pathway are the important components of the segmentation clock [3]. In particular, the Notch and Wnt pathways regulate the oscillating expressions of their target genes, which play major roles in controlling somite formation [4-6]. In recent years mathematical models have been proposed to reveal the mechanisms of the two pathways and their crosstalk in the process of somitogenesis. In 2003 Julian Lewis et al. proposed a simple mathematical model of the Notch pathway in zebrafish somitogenesis [7]. They modeled the oscillating expressions of Notch pathway target genes by introducing two feedback loops. In 2009, Smita Agrawal et al. proposed a model of the Notch pathway during somitogenesis to elucidate the mechanisms of context-dependent signaling of the Notch pathway [8]. They modeled bistability in Notch signaling. In 2010 Alan J. Terry et al. proposed a spatio-temporal model of Notch signaling in the zebrafish segmentation clock [9]. They adopted a spatially-explicit modeling approach that can display intracellular protein diffusion graphically. In the same year, Peter B. Jensen et al. proposed a mathematical model to capture the oscillation of the Wnt pathway in somitogenesis [10]. The core of their model was a negative feedback loop centered on Axin2. Now, more and more evidence supports the view that somite formation relies on complex cooperation among multiple signaling pathways. In 2007, J.G. Rodríguez-González et al. proposed a mathematical model to investigate the interaction between the Notch and the Wnt pathways in the segmentation clock in mice [11]. In 2008, Albert Goldbeter et al. proposed a theoretical model for understanding the mechanism of interactions among the Notch, Wnt and FGF pathways [12]. Moirés Santillán et al. also proposed a mathematical model for the gene regulatory network of the mouse embryo to elucidate somite formation [13]. In 2009, A. Kazama et al. proposed a mathematical model to reveal the interaction of the Notch and Wnt pathways in the segmentation clock [14]. Although the simulation results of these models agree well with some results of biological experiments, they only considered simple interaction relationships between the two pathways. More accurate and complicated mathematical models are still needed to further our understanding of the detailed mechanism of the Notch and Wnt pathways and their crosstalk in somitogenesis.

In this study, we have proposed three more complicated mathematical models for the Notch and Wnt signaling pathways and their crosstalk in somitogenesis, taking the mouse as example. In modeling the Notch and Wnt signaling pathways in isolation, three core negative feedback loops centered on Lfng, Hes7 and Axin2, respectively, were considered, while in the combined model of the two pathways, three levels of cross-regulation were modeled. These models not only simulate the periodic expressions of the Notch and Wnt target genes in somitogenesis, but also reproduce the wet experimental results in the literature. The simulations demonstrate that the extracellular Notch and Wnt signals are essential for the oscillating expressions of both Notch and Wnt target genes. Moreover, the internal negative feedback loops and the three levels of crosstalk between the Notch and Wnt pathways play important but distinct roles in maintaining the system oscillation. In addition, the results of the parameter sensitivity analysis of the models indicate that the Notch pathway is more sensitive to perturbation in somitogenesis.

## Results

### The model for the Notch pathway in isolation

Notch-mediated signaling is initiated via the binding of the delta-like 1 (Dll1) ligand to the Notch receptor. Then the intracellular domain of Notch (NICD) is cleaved from the membrane tether. It is transported into the nucleus and associates with the recombining binding protein (RBP-j) to form a transcriptional activator that activates the transcription of a set of target genes, including the Lunatic Fringe (Lfng) and the hairy and enhancer of split 7 (Hes7) genes. The Lfng and Hes7 mRNAs are transported from nucleus and are translated into proteins in the cytoplasm. Lfng inhibits the cleavage of NICD from Notch leading to repression of the transcription factor NICD/ RBP-j, so a negative feedback loop in the Notch pathway, which is termed “the big feedback loop”, is formed [15]. Hes7 inhibits the transcription of both itself and the Lfng gene, and thus another negative feedback loop of the Notch pathway, termed “the small feedback loop”, is formed [16]. The periodic expressions of Lfng and Hes7 genes are essential for somite formation [5].

**Figure 1:**
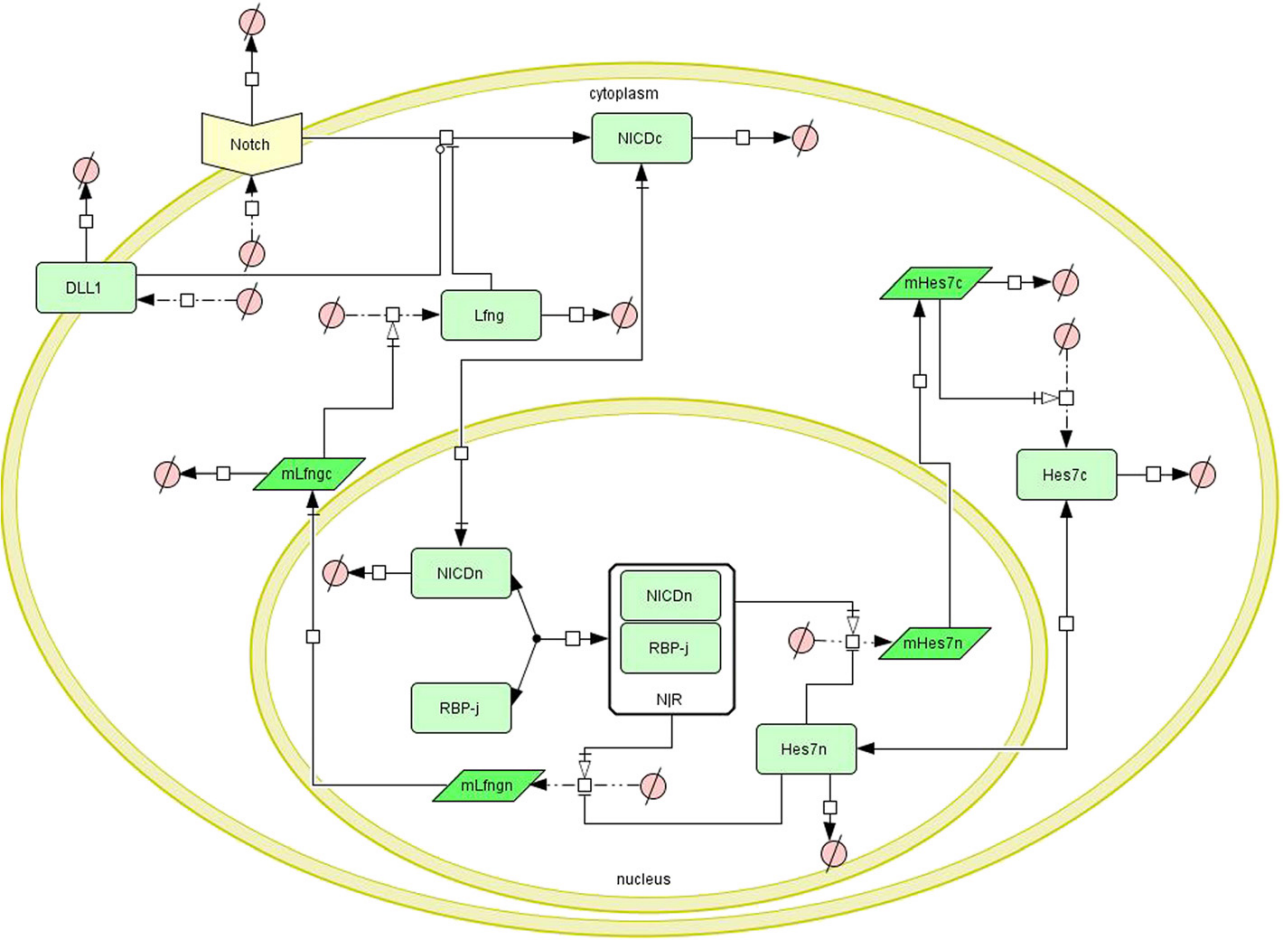
**Schematic diagram of the Notch signaling pathway.** The diagram was created using CellDesigner. The light green rectangle represents protein; the bottle green parallelogram represents mRNA; the white rectangle that contains a light green rectangle represents a complex; ∅ represents the resultant of a degradation reaction or reactant of a combination reaction. The arrow represents the reaction.

On the basis of the above analysis, a mathematical model for the Notch signaling pathway was established. A schematic diagram of this model is given in Figure 1. In the modeling process, the following hypotheses were proposed: A cell is divided into two compartments, the nucleus where target genes are transcribed and the cytoplasm where proteins are translated. Transcription factors such as NICD and Hes7 can shuttle between the nucleus and cytoplasm and degrade in both compartments; mRNA molecules only can be transported from the nucleus to the cytoplasm and degrade there. These hypotheses are applicable to all three models in the present study. A total of 12 ordinary differential equations (ODEs) for the Notch signaling model and their biological explanations are given in Additional file 1. Here we assumed the Dll1 ligand and the Notch receptor are synthesized at a constant rate and the degradation of these molecules obeys Michaelis-Menten kinetics. Two points are noteworthy: (1) mRNAs are only degraded in the cytoplasm. (2) RBP-j is not degraded because we assume the total concentration of RBP-j remains constant. In particular, we modeled the big feedback loop centered on Lfng as four major reactions. The first reaction is the cleavage of NICD from Notch under the condition of activation of the Dll1 ligand, which is represented using a mass action equation because the rate of NICD synthesis is proportional to the concentration of the Notch receptor, while the activation of Dll1 to the cleavage of NICD is represented using a Hill equation with Hill coefficient 1 because Dll1 catalyzes Notch at only one site; also, the inhibition of the cleavage of NICD by Lfng is represented using a Hill equation with Hill coefficient −2 because Lfng binds to Notch at two sites. (This reaction is represented in Eq 1.3 in Additional file 1). The second reaction is the reversible binding of NICD to RBP-j in the nucleus thereby forming a transcriptional activator. We modeled this reaction using a mass action equation (Eq 1.9 in Additional file 1). The third reaction is the transcription of the Lfng gene in the nucleus under the activation of NICD-RBP-j activator and the repression of the Hes7 protein. We modeled the active regulation of the NICD-RBP-j activator using a Hill equation with Hill coefficient 2 and the repressive regulation of Hes7 using a Hill equation with Hill coefficient −2 (Eq 1.11 in Additional file 1). The fourth reaction is the translation of Lfng mRNA in the cytoplasm, which is modeled using a mass action equation (Eq 1.4 in Additional file 1). The small feedback loop centered on Hes7 is modeled as three major reactions: The first is the transcription of the Hes7 gene in the nucleus under the activation of NICD-RBP-j activator and the repression of the Hes7 protein (Eq 1.12 in Additional file 1). The second is the shuttling of the Hes7 protein between cytoplasm and nucleus, which is modeled using a mass action equation (Eq 1.10 in Additional file 1). The third is the translation of Hes7 mRNA, which is also modeled using a mass action equation (Eq 1.6 in Additional file 1).

### Model simulation for the Notch pathway in isolation

**Figure 2:**
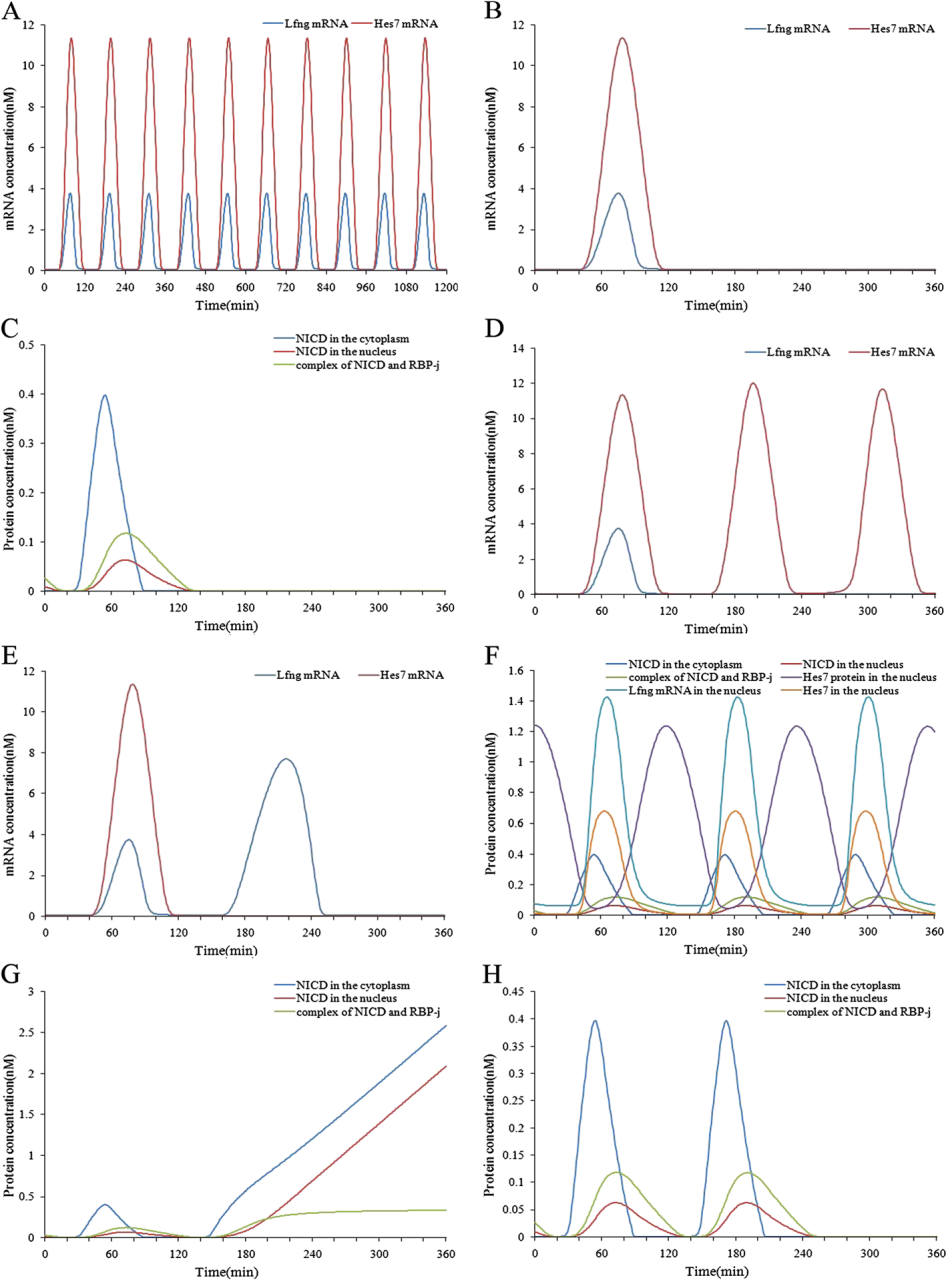
**Simulation results of the Notch signaling model.** (**A**) The oscillating expressions of Notch target genes under conditions of a constant extracellular signal. (**B**) The expression patterns of Notch target genes after the Dll1 gene was knocked out at time point 120 minutes. (**C**) The changes of concentration of NICD in the cytoplasm and nucleus and the transcriptional activator after Dll1 was knocked out at time point 120 minutes. (**D**) The expression patterns of the Hes7 gene after the Lfng gene was knocked out at time point 120 minutes. (**E**) The expression patterns of the Lfng gene after the Hes7 gene was knocked out at time point 120 minutes. (**F**) The phase relationships of the Notch target genes, Hes7 and NICD. (**G**) The changes of concentration of NICD after the Lfng gene was knocked out at time point 120 minutes. (**H**) The changes of concentrations of NICD and the complex of NICD and RBP-j after the Hes7 gene was knocked out at time point 120 minutes.

The Notch pathway in somitogenesis of the animal embryo is an oscillating system. Its target genes are expressed in a period of about 120 minutes in the mouse, 90 minutes in the chicken and 30 minutes in the zebrafish, which are synchronous with the formation of the somite [17]. All our models take mouse as example, and thus the oscillating period of target genes is taken as 120 minutes. It is easy to change the period of the models by changing the training set of the parameter learning algorithm to adapt the model to other applications. The cyclic expressions of Notch pathway target genes drive the mature cells traveling from the rostral to the caudal end in the PSM during the formation of one somite. So the periodic expressions of target genes are crucial to somitogenesis. The simulated expression patterns of the Notch target genes under conditions of a constant extracellular signal are illustrated in Figure 2 (A). From the figure, we can see that the target genes of the Notch pathway are expressed in a cyclic manner, and the oscillating period is about 120 minutes; all its target genes are in phase. So the model can simulate the dynamics of the Notch signaling pathway.

We used this model to perform simulations and tried to reveal the molecular mechanism behind the phenomena. First, we investigated the influence of the upstream Notch signals on the expressions of the target genes. When knocking out the Dll1 gene, the ligand of the Notch pathway, at time point 120 minutes, we found the expressions of the Notch target genes do not oscillate and the expression levels descend markedly (see Figure 2 (B)). This suggests that Dll1 is essential for the normal expressions of Notch target genes. When knocking out the Notch gene, receptor of the Notch pathway, at time point 120 minutes, the oscillating expressions of the target genes disappear as in the knockout of Dll1 (see Figure 2 (B)). Moreover, knockout of Dll1 or Notch made the expressions of NICD and the NICD-RBP-j transcriptional activator disappear (see Figure 2 (C)). NICD is the direct regulator of the Notch pathway target genes, so when it disappeared the oscillating expressions of the target genes were destroyed. The results demonstrate that the activity of upstream Notch signals is necessary for the oscillating expressions of the Notch pathway target genes.

Next, we investigated the influence of the feedback loops on the oscillating expressions of the Notch pathway target genes. After knocking out the Lfng gene at time point 120 minutes, we found that expression of Hes7 gene still oscillated, though its maximum expression level increased a little (see Figure 2 (D)). This suggests that the big negative feedback loop formed by Lfng is not essential for the oscillating expressions of the Notch pathway target genes. Similarly, we knocked out the Hes7 gene after one period. It was found that the expression of the Lfng gene increased markedly and its oscillating expression was destroyed (see Figure 2 (E)). This suggests that the small negative feedback loop formed by Hes7 is necessary for the oscillating expressions of the Notch pathway target genes. NICD in the big feedback loop induced the expressions of the target genes, whereas Hes7 in the small feedback loop inhibited them, so the oscillating pattern of the target genes was in phase with NICD but in antiphase with Hes7 (see Figure 2 (F)). The phase relationship of these two feedback loops in the Notch pathway is crucial for the correctly oscillating expressions of the target genes. After knocking out the Lfng gene, NICD increased quickly but the NICD-RBP-j transcriptional activator only increased a little owing to the constant concentration of RBP-j (see Figure 2 (G)). The expression of Hes7 then increased a little with the NICD-RBP-j transcriptional activator, but the concentration increase of NICD did not influence the oscillating expression of Hes7 (see Figure 2 (G)). On the other hand, after Hes7 is knocked out, NICD and the NICD-RBP-j transcriptional activator did not change immediately, but decreased to nearly zero after about one period (see Figure 2 (H)). Obviously, the increase of Lfng gene expression is not due to the concentration increase of its transcriptional activator (the NICD-RBP-j complex) but to the concentration decrease of its inhibitor (Hes7). The delayed concentration decrease of NICD and the NICD and RBP-j transcriptional activator was mainly due to a large increase in Lfng after Hes7 was knocked out, which precluded the cleavage of NICD from Notch. The influence of Hes7 on upstream Notch signals is not instantaneous but follows a time delay. Therefore, although the two negative feedback loops are all-important for somitogenesis, the small feedback loop is closely related to the periodic expressions of the Notch pathway target genes, while the big feedback loop is complementary to the periodic expressions of those genes. However, the big feedback loop is essential for the oscillating expressions of the Wnt pathway target genes in the combined model (see the section describing the combined model). Research of Ferjentsik et al. indicated that the big feedback loop is important for the formation of the somite in mouse embryo development [18]. In their study, after the Lfng gene of the mouse embryo was knocked out, Notch activity was still dynamic but the somite was irregular in these embryos.

In summary, the simulation results demonstrate that the model for the Notch signaling pathway in isolation is capable of simulating the oscillating expressions of the Notch pathway target genes, and can also reproduce the wet experimental results. It has the potential for further use in research on the molecular mechanism of somitogenesis.

### The model for the Wnt signaling pathway in isolation

**Figure 3:**
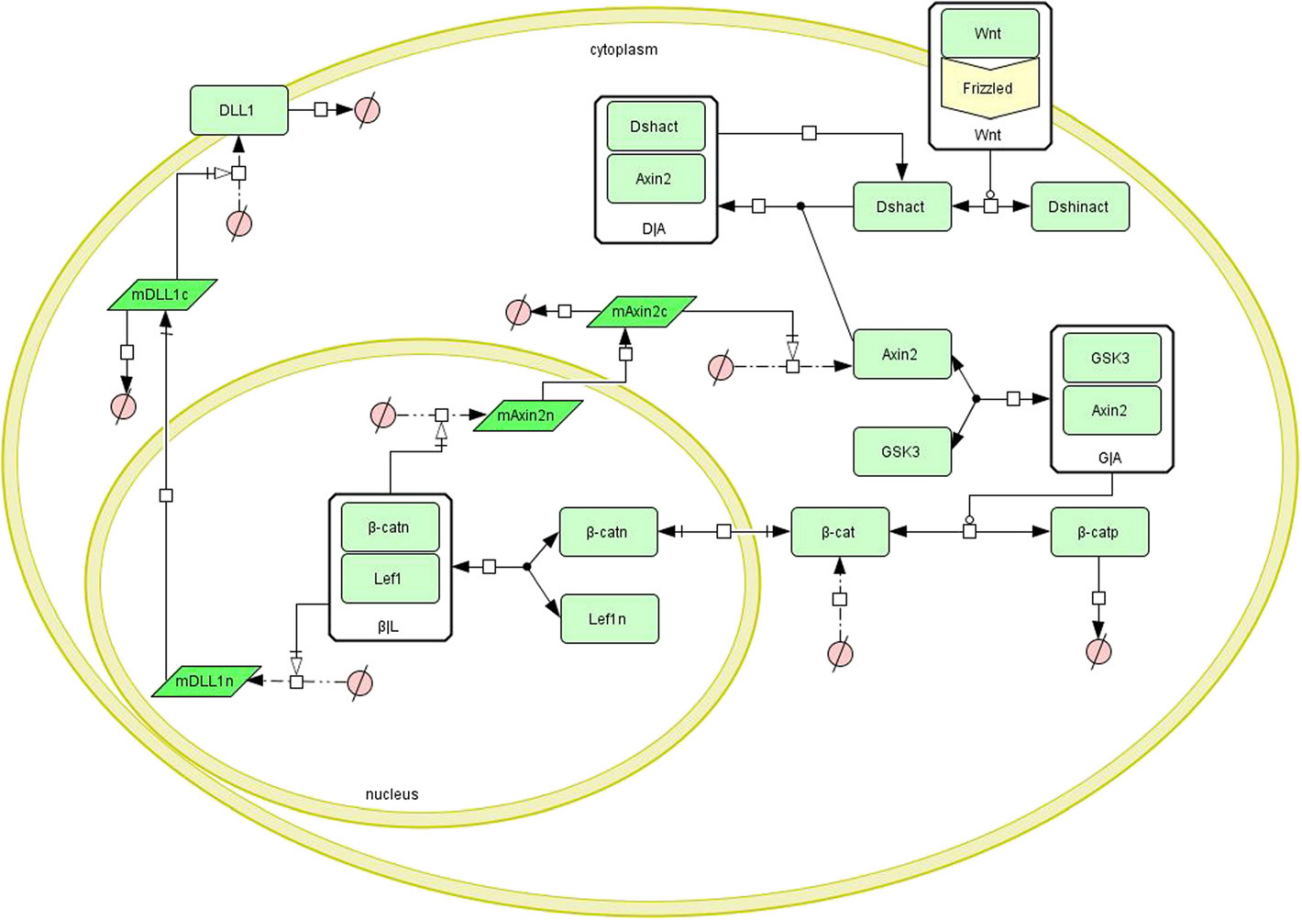
**Schematic diagram of the Wnt signaling pathway.** The diagram was created using CellDesigner. The light green rectangle represents protein; the bottle green parallelogram represents mRNA; the white rectangle that contains a light green rectangle represents a complex; ∅ represents the resultant of a degradation reaction or reactant of a combination reaction. The arrow represents the reaction.

A schematic diagram of the Wnt signaling pathway is presented in Figure 3. First the Wnt ligand binds to its receptor and activates the dishevelled (Dsh) protein. Then the active Dsh recruits the axis inhibition protein 2 (Axin2) from the degradation complex and then destroys it. The degradation complex consists of Axin2, glycogen synthase kinase 3 (GSK3) and β-catenin, which is the crucial regulator of the Wnt pathway. The β-catenin in the degradation complex is phosphorylated and degraded quickly. So the concentration of dissociated β-catenin in the cytoplasm is very low without Wnt signals. Following the recruitment of Axin2 by the active Dsh, the degradation complex is destroyed and the β-catenin is liberated [19]. With the increase of the dissociated β-catenin, a pool of β-catenin is formed in the cytoplasm. It is transported into the nucleus and associates with Lymphoid enhancer-binding factor-1 (Lef1) to form a transcriptional activator which activates the transcription of a set of target genes, including Axin2 and Lef1 [20]. Axin2 is an essential target gene of the Wnt pathway, because it in turn inhibits Wnt signals by degrading β-catenin, such that a negative feedback loop is formed. Lef1 is an important transcription factor, and is also a downstream target gene of the Wnt pathway [21]. Moreover, many studies have indicated that Dll1, ligand of the Notch pathway, is also a downstream gene of the Wnt pathway [22,23].

A total of 13 ODEs for the Wnt signaling model and their biological explanations are given in Additional file 1. We assumed the activation of Dsh is reversible and obeys Michaelis-Menten kinetics. The catalysis of Dsh activation by Wnt was modeled using a Hill equation with Hill coefficient 1 (Eq 2.1 in Additional file 1). The transcription of the Dll1 gene in the nucleus when the β-catenin-Lef1 complex is activated was modeled using a Hill equation with Hill coefficient 2 (Eq 2.13 in Additional file 1), and the translation of the Dll1 mRNA was modeled using a mass action equation (Eq 2.7 in Additional file 1). The negative feedback loop centered on Axin2 was modeled using six major reactions. A mass action equation was used to model the reversible binding of GSK3 to Axin2 to form the degradation complex that phosphorylates β-catenin (Eq 2.3 in Additional file 1). The reversible phosphorylation of β-catenin when the GSK3-Axin2 complex acts as catalyst was modeled using a Michaelis-Menten equation with the catalysis rate proportional to the GSK3 of the GSK3-Axin2 complex in the total GSK3 (Eq 2.6 in Additional file 1). The two above reactions guarantee a very low concentration of the unphosphorylated β-catenin in the cytoplasm so as not to initiate the expressions of the Wnt pathway target genes, including the Axin2 gene. The binding of active Dsh to Axin2 and degradation of Axin2 were modeled using a mass action equation (Eq 2.2 in Additional file 1). This reaction can destroy the degradation complex by degrading Axin2, so that the unphosphorylated β-catenin protein can enter the nucleus to activate target genes. The binding of unphosphorylated β-catenin to Lef1 in the nucleus to form the transcription activator was modeled using a mass action law (Eq 2.11 in the Additional file 1). The transcription of the Axin2 gene when the β-catenin-Lef1 complex is activated was modeled using a Hill equation with Hill coefficient 2 because the complex binds to the Axin2 gene at 2 sites (Eq 2.12 in Additional file 1). The sixth reaction is the translation of the Axin2 mRNA, which was modeled using a mass action equation on the assumption that the translation rate is proportional to the Axin2 mRNA concentration (Eq 2.4 in Additional file 1).

### Model simulation for the Wnt pathway in isolation

**Figure 4:**
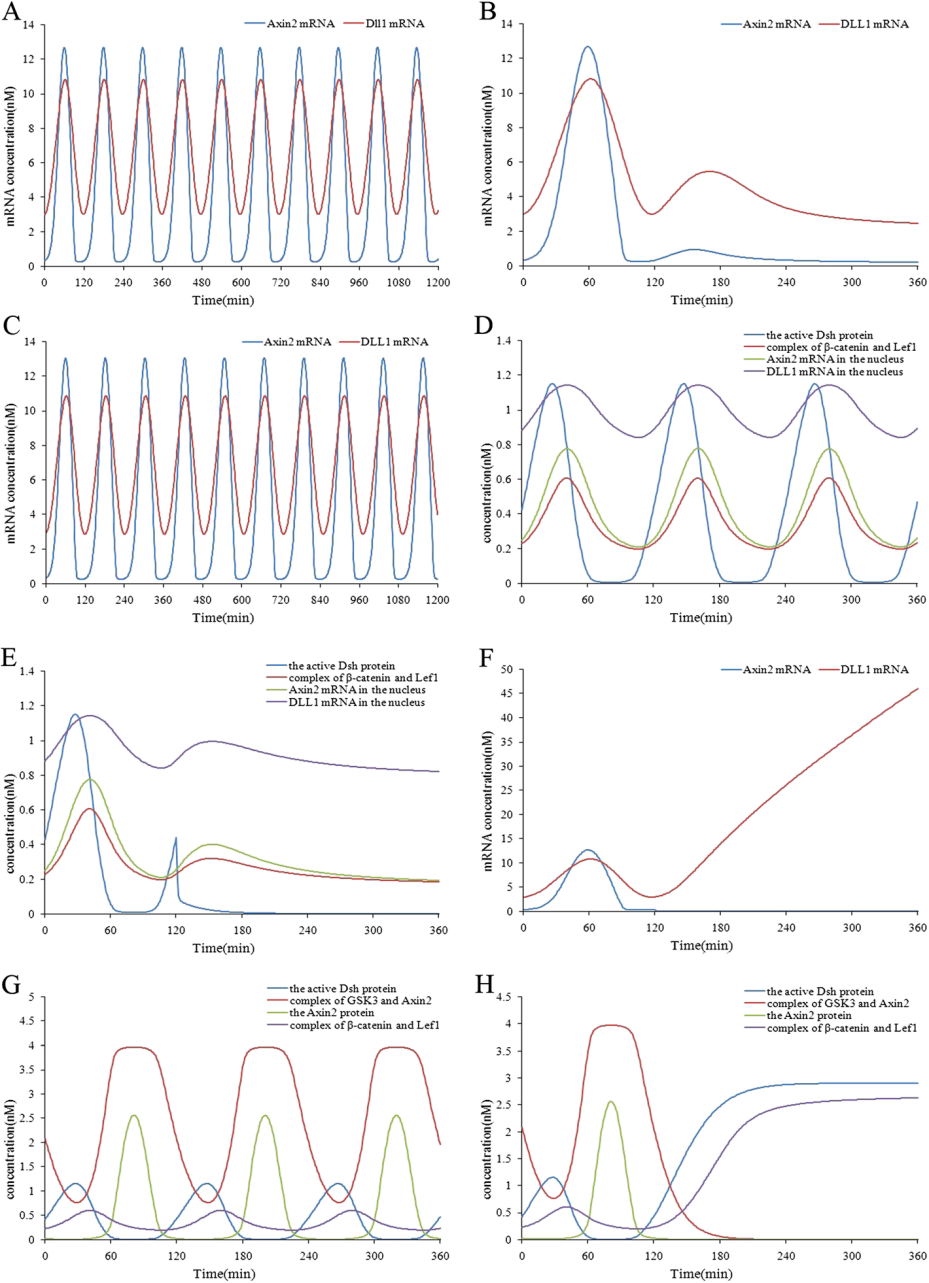
**Simulation results of the Wnt signaling model.** (**A**) The oscillating expressions of Wnt target genes under conditions of a constant extracellular signal. (**B**) The expression patterns of Wnt target genes after the extracellular Wnt signals were removed at time point 120 minutes. (**C**) The expression patterns of the Wnt target genes after the extracellular Wnt signals were doubled. (**D**) The synchronous expressions of Wnt target genes with the downstream Wnt signals and the synchronously delayed expressions between the downstream and upstream Wnt signals. (**E**) The changes of concentration of active Dsh and the β-catenin- Lef1 complex and the expression patterns of the Wnt target genes after the extracellular signals were knocked out at time point 120 minutes. (**F**) The expression patterns of the Dll1 gene after the Axin2 gene was knocked out at time point 120 minutes. (**G**) The phase relationships between active Dsh, the GSK3-Axin2complex, the β-catenin-Lef1 complex and Axin2. (**H**) The changes of concentration of Axin2, active Dsh, the GSK3-Axin2 complex and the β-catenin-Lef1 complex after the Axin2 gene was knocked out at time point 120 minutes.

It is known that the Wnt pathway in somitogenesis is also an oscillating system, but its target genes are expressed in antiphase with the Notch pathway [6]. The simulated expression patterns of Wnt target genes when there is a constant extracellular signal are presented in Figure 4 (A). From the figure, we can see that the target genes of the Wnt pathway are expressed in a cyclic manner, and the oscillating period is about 120 minutes; all its target genes are in phase. So the model can simulate the dynamics of the Wnt signaling pathway. We used this model to perform simulations and tried to reveal the molecular mechanism behind the phenomena. First, we investigated the influence of the extracellular Wnt signals on the expressions of the target genes. When the extracellular Wnt signals were removed at time point 120 minutes, we found that the oscillating expressions of the Wnt pathway target genes disappeared and their expressions descended monotonically (see Figure 4 (B)). When the concentration of the extracellular Wnt signals was doubled, we found that the expression levels and oscillating period of the target genes were not affected (see Figure 4 (C)). We chose Dsh as the representative of the upstream Wnt signals owing to its direct activation by Wnt signals, and the β-catenin-Lef1 transcriptional activator as the representative of the downstream Wnt signals because it is the direct regulator of the target genes. As illustrated in Figure 4 (D), the expressions of the target genes were synchronous with the downstream Wnt signals but were later than the upstream signals. After knocking out the extracellular Wnt signals at time point 120 minutes, we found the activity of Dsh immediately disappeared and the downstream Wnt signals decreased after a while (see Figure 4 (E)). The simulation results show that the extracellular Wnt signals are essential for the synchronous oscillation of the network, but increasing Wnt signals do not disturb the oscillating period of the target genes. This is in accord with the findings of Sarah Gibb et al. [4]. Despite time delays, the expressions of the target genes and the upstream and downstream signals are still in phase. Next, we researched the influence of the feedback loop formed by Axin2 on the expressions of the target genes. The simulation result of knocking out the Axin2 gene at time point 120 minutes is presented in Figure 4 (F). From the figure, we can see that the Dll1 gene was up-regulated and ceased to oscillate after the Axin2 gene was knocked out. Axin2 is an important component of the negative feedback loop, so its knockout seriously disturbed the oscillating expressions of Wnt target genes. This is again in accord with the experimental findings of Sarah Gibb et al. [4]. The phase relationship of the components in the feedback loop can be seen in Figure 4 (G). The active Dsh is in antiphase with the GSK3-Axin2 degradation complex. It recruits Axin2 in a competitive manner from the GSK3-Axin2 degradation complex and thereby destroys it. So when the active Dsh increases the GSK3-Axin2 degradation complex decreases, and vice versa. The GSK3-Axin2 degradation complex is in antiphase with the β-catenin-Lef1 transcriptional activator, because it degrades β-catenin and thus inhibits the formation of the β-catenin-Lef1 complex. The active Dsh is in antiphase with Axin2, because Axin2 inhibits the activity of Dsh by binding to it. The GSK3-Axin2 degradation complex is in phase with Axin2. From the above observations, we conclude that the active Dsh and the β-catenin-Lef1 transcriptional activator in the feedback loop are the activators of the target genes, whereas Axin2 and the GSK3-Axin2 degradation complex are the inhibitors of them. These components regulate the correct expressions of Wnt target genes so they cooperate with each other. We further researched the concentration changes of the components in the Wnt pathway when the Axin2 gene was knocked out (see Figure 4 (H)). It was found that Axin2 knockout resulted in increase of active Dsh and disappearance of the GSK3-Axin2 degradation complex. As a result, the concentration of β-catenin and Lef1 ascended monotonically. As a result, the Wnt pathway ceased to oscillate.

In summary, the simulation results demonstrate that the model for the Wnt signaling pathway in isolation is capable of simulating the oscillating expressions of the Wnt pathway target genes, and also reproduces the wet experimental results. It has the potential for use in further research on the molecular mechanism of somitogenesis.

### The combined model for crosstalk between the Notch and Wnt pathways

The crosstalk between the Notch and Wnt pathways in somitogenesis is very complicated. Some biological experiments have confirmed several different levels of crosstalk. The first level is through Dll1, which is the ligand of Notch pathway and is also a downstream target gene of the Wnt pathway. Research by Juan Galceran et al. [22] confirmed that Lef1 binds to β-catenin in the nucleus to form the Lef1-β-catenin complex that activates the expression of endogenous Dll1 in fibroblasts. Research by Michael Hofmann et al. [23] indicated that the Lef/Tcf factors, which are regulators of the Wnt pathway, activate transcription of Dll1 cooperating with TBX6. The second level is through NICD in the Notch pathway and Dsh in the Wnt pathway, respectively. Studies by Alexander Aulehla et al. [6] indicated that NICD binds to active Dsh and thus inhibits the Wnt pathway; meanwhile, Notch signals are also inhibited. Moreover, the oscillating expressions of the Notch pathway target genes are in antiphase with the Wnt pathway target genes, so it is conjectured that the two pathways inhibit each other alternately. There is molecular evidence supporting this viewpoint. In Drosophila, it was found that NICD binds to the PDZ domain of Dsh, while Dsh interacts antagonistically with NICD [24]. It is likely that Dsh blocks Notch signaling directly through binding of NICD [24]. JG Rodríguez-González et al. [11] established a mathematical model to simulate this level of crosstalk between the two pathways. Their model simulates the results of Alexander Aulehla et al. [6]. The third level is through naked cuticle 1 (Nkd1) protein, which is encoded by a Wnt downstream gene. Research by Aki Ishikawa et al. [25] indicated that the transcription of Nkd1 was extremely downregulated in the PSM of vestigial tail (vt/vt), a hypomorphic mutant of Wnt3a, so it can be speculated that the Nkd1 gene is regulated by Wnt signals. Moreover, Nkd1 oscillation had a similar phase to Lfng transcription and they were arrested in the Hes7 mutation embryo, hence it is likely that Nkd1 is regulated by Hes7. Furthermore, Nkd1 inhibits Wnt signals by binding to Dsh.

**Figure 5:**
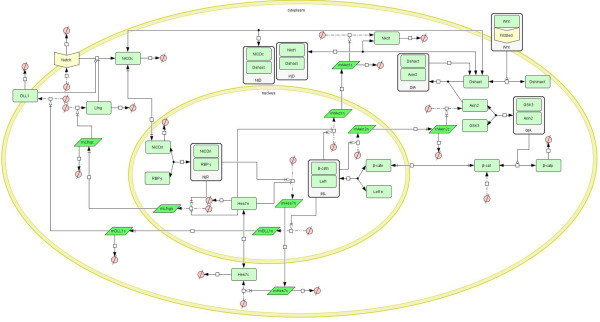
**Schematic diagram of the crosstalk between the Wnt and Notch pathways.** The diagram was created using CellDesigner. The light green rectangle represents protein; the bottle green parallelogram represents mRNA; the white rectangle that contains a light green rectangle represents a complex; ∅ represents the resultant of a degradation reaction or reactant of a combination reaction. The arrow represents the reaction.

On the basis of the above experimental observations and the models of the single Notch and Wnt pathways described in the previous sections, we established a combined model of the two pathways by introducing the three levels of crosstalk. A schematic diagram of the combined model is presented in the Figure 5. Besides the modification to some ODEs in the isolated Notch and Wnt pathway models, we added five new ODEs in the combined model. Detailed explanations of the modified and added ODEs are given in Additional file 1. Dll1 is the ligand of the Notch pathway and it is also a downstream target gene of the Wnt pathway. It has been modeled using a mass action equation in the Wnt signaling model (Eq 2.7 in Additional file 1). At the same time, in the Notch signaling model, it was assumed that Dll1 is synthesized at a constant rate (Eq 1.1 in Additional file 1). So Eq 2.7 was preserved, while Eq 1.1 was removed in the combined model. In the second level of crosstalk, we modeled the reversible binding of NICD to active Dsh through a mass action equation (Eq 3.1 in Additional file 1). In the third level of crosstalk, we introduced six new reactions to model the expression and functioning of Nkd1. The transcription of the Nkd1 gene in the nucleus under the activation of the β-catenin-Lef1 complex and the repression of the Hes7 protein was modeled using two Hill equations with Hill coefficients 2 and −2, respectively (Eq 3.3 in Additional file 1). The transport of Nkd1 mRNA from the nucleus to the cytoplasm was modeled by a mass action equation because the transport rate is proportional to the concentration of Nkd1 mRNA in the nucleus (Eq 3.4 in Additional file 1). The translation of Nkd1 mRNA in the cytoplasm was modeled by a mass action equation (Eq 3.5 in Additional file 1). The reversible binding of Nkd1 to active Dsh, thereby inhibiting the Wnt pathway, was modeled by a mass action equation (Eq 3.6 in Additional file 1). Lastly, the degradation of the Nkd1 mRNA and protein was modeled using Michaelis-Menten equations in the cytoplasm (Eq 3.4 and Eq 3.5 in Additional file 1).

### Model simulation for crosstalk between the Notch and Wnt pathways

**Figure 6:**
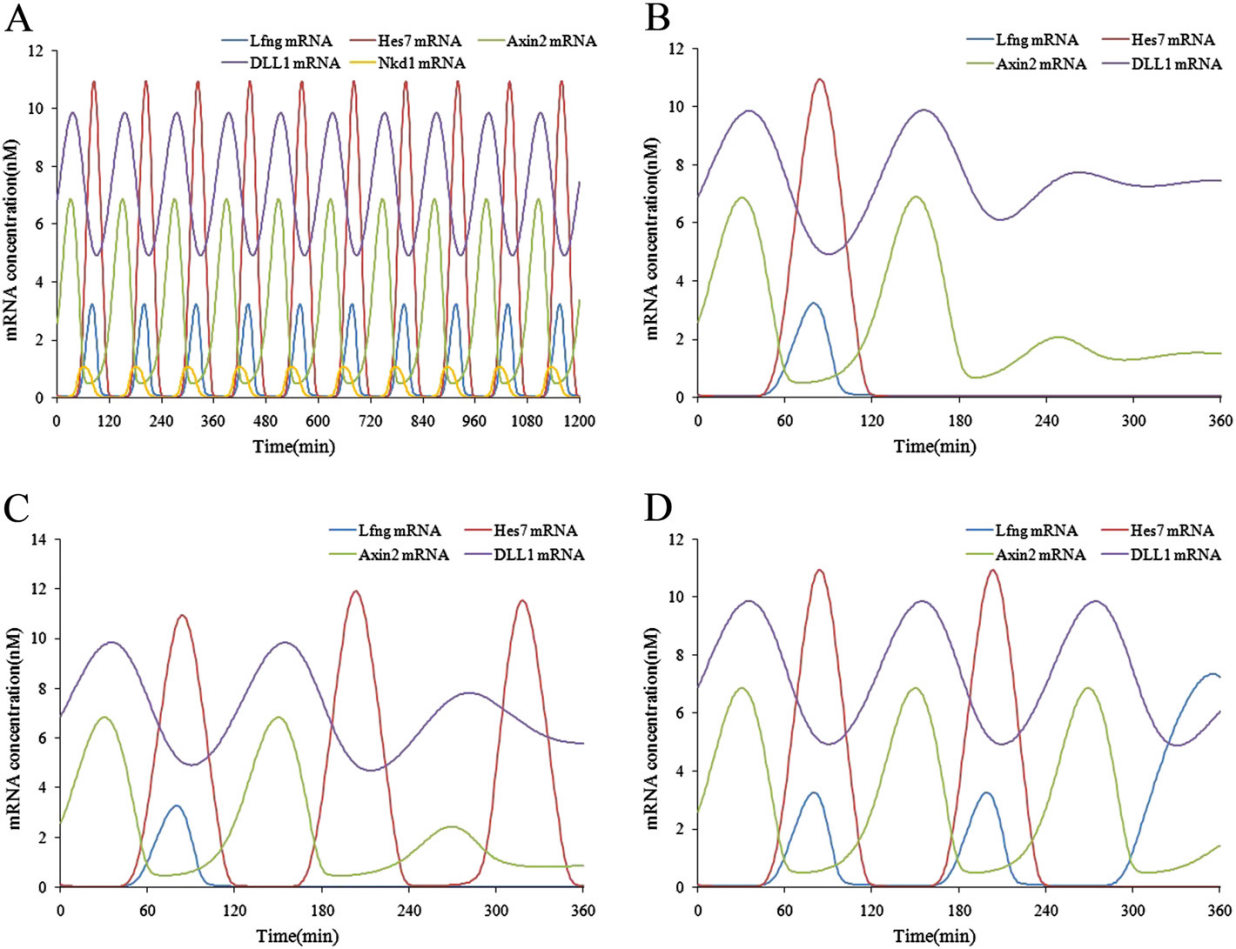
**Simulation results of the combined model under conditions of normal extracellular signals and knocking out components of the Notch pathway.** (**A**) The oscillating expressions of Notch and Wnt target genes under conditions of a constant extracellular Wnt signal. (**B**) The expression patterns of the Notch and Wnt target genes after the Notch gene was knocked out at time point 120 minutes. (**C**) The expression patterns of the Hes7 gene and the Wnt target genes after Lfng was knocked out at time point 120 minutes. (**D**) The expression patterns of the Lfng gene and the Wnt target genes after the Hes7 gene was knocked out at time point 120 minutes.

Under the condition of a constant Wnt ligand concentration, the simulation result of the combined model is presented in Figure 6 (A). From the figure, we can see that the target genes of both the Notch and Wnt pathways showed oscillating expression patterns. Among the five target genes in the model, Lfng, Hes7 and Nkd1 are in phase, whereas Axin2 and Dll1 are in antiphase with the former. Moreover, the oscillating period of all these genes is about 120 minutes. So the combined model simulates the dynamics of both the Notch and Wnt signaling pathways. When the Notch gene is knocked out at time point 120 minutes, the Notch pathway target genes are not expressed as in the simulation result from the isolated Notch model, and the expression levels of the Wnt target genes became lower and ceased to oscillate after a time delay (see Figure 6 (B)). This is in accord with the research of Sarah Gibb et al. [4]. In their research, when the Notch signal was abolished pharmacologically, the Notch target genes were not expressed and the Axin2 gene was downregulated. When the Lfng gene was knocked out at time point 120 minutes, the maximum concentration of Hes7 increased but was still oscillating as in the isolated Notch model, whereas Wnt target genes were downregulated after a time delay of about 30 minutes and ceased to oscillate (see Figure 6 (C)). Lfng inhibits the cleavage of NICD from the Notch receptor, so knocking out of the Lfng gene led to an increase of NICD. As a result, the transcription of the Hes7 gene was enhanced. On the other hand, NICD binds to Dsh and inhibits Wnt signals, so when the concentration of NICD increased, Wnt signals decreased, and consequently the target genes of the Wnt pathway were downregulated and lost their oscillation. When the Hes7 gene was knocked out after two periods, we found that Lfng was upregulated as in the isolated Notch model, but the expressions of the Wnt pathway target genes were not influenced (see Figure 6 (D)). Hes7 inhibits the transcription of Lfng, so knocking out the Hes7 gene enhanced the expression of Lfng, but there was no obvious change in the concentration of NICD or the expression of the Dsh gene. As a result, the Wnt target genes were not influenced. The above simulation results involving two feedback loops were confirmed by the research of Zoltan Ferjentsik et al. [18].

From our simulation experiments, we are able to draw some interesting conclusions: The big feedback loop centered on Lfng in the Notch pathway is complementary to the periodic expressions of the Notch target genes, but is essential for the oscillating expressions of the Wnt target genes in the combined model. In contrast, the small feedback loop centered on Hes7 in the Notch pathway is essential for the oscillating expressions of the Notch target genes, but is not related to the oscillating expressions of the Wnt target genes in the combined model.

**Figure 7:**
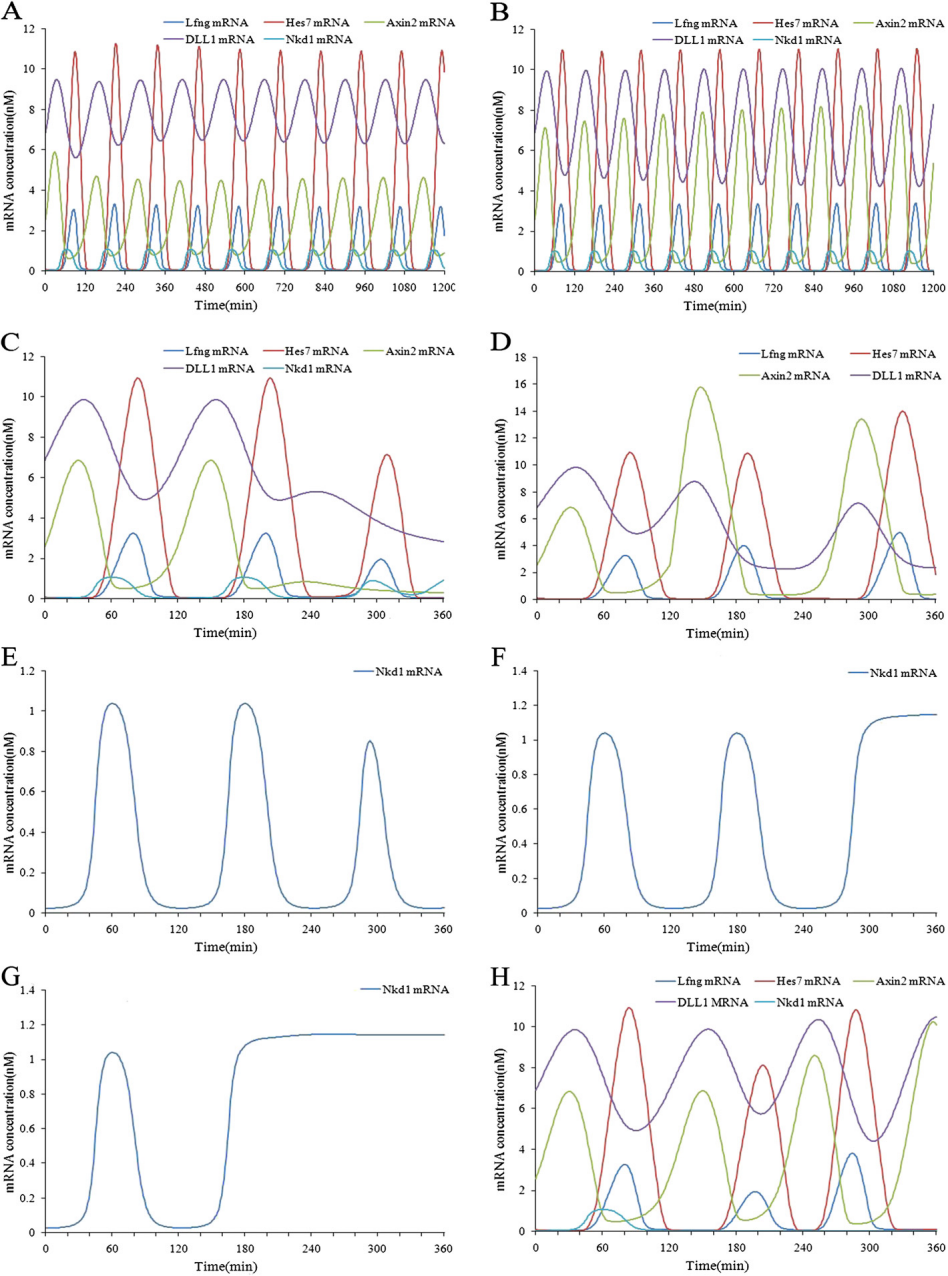
**Simulation results of the combined model under conditions of knocking out components of the Wnt pathway and the Nkd1 gene.** (**A**) The changes in gene-expression oscillation periods of the Wnt and Notch pathways after Wnt signals were reduced. (**B**) The changes in gene-expression oscillation periods of the Wnt and Notch pathways after Wnt signals were increased 10-fold. (**C**) The expression patterns of the target genes of the Wnt and Notch pathways after Wnt signals were knocked out at time point 200 minutes. (**D**) The expression patterns of the target genes of the Wnt and Notch pathways after the Axin2 gene was overexpressed at time point 120 minutes. (**E**) The expression patterns of the Nkd1 gene after Wnt signals were knocked out at time point 240 minutes. (**F**) The expression patterns of the Nkd1 gene after the Dll1 gene was knocked out at time point 240 minutes. (**G**) The expression patterns of the Nkd1 gene after the Hes7 gene was knocked out at time point 120 minutes. (**H**) The expression patterns of the target genes of the Wnt and Notch pathways after Nkd1 was knocked out at time point 120 minutes.

Next, we investigated the regulation of the Notch pathway by the Wnt pathway. When the Wnt signals were halved, we found that the number of periods of the target genes in both the Notch and Wnt pathways was fewer than ten within simulation time intervals of 1200 minutes, i.e. the oscillating period of the target genes was lengthened (more than 120 minutes) (see Figure 7 (A)). However, when we upregulated the Wnt signals 10-fold, the period of the oscillating expressions of the target genes was not obviously changed (see Figure 7 (B)). The above simulation results are supported by the findings of Sarah Gibb et al. [4]. Lastly, we knocked out the Wnt signals at time point 200 minutes. The target genes of the Wnt and Notch pathways were all downregulated and ceased to oscillate (see Figure 7 (C)). This is because Wnt signals are regulators of Dll1. Removing Wnt signals led to the downregulation of Dll1 expression and consequently resulted in Notch signals decreasing to zero. These results are in accord with the findings of Alexander Aulehla et al. [6]. Therefore, Wnt signals influence the expressions not only of Wnt target genes, but also of Notch target genes. We next researched the influence of the feedback loop in the Wnt pathway on the expressions of the target genes of the Notch and Wnt pathways. When the Axin2 gene was knocked out, we found that the Dll1 gene was upregulated and its expression failed to oscillated, whereas the Notch target genes were still oscillating (result not shown). Lastly, we overexpressed the Axin2 gene at time point 120 minutes. We found that the Dll1 gene was downregulated, while the Notch target genes, especially the Lfng gene, were upregulated, but they still remained oscillating (see Figure 7 (D)). These simulation results are in accord with the experiments of Alexander Aulehla et al. [6]. Therefore, extracellular Wnt signals are essential for the oscillating expressions of the Wnt and Notch target genes. The feedback loop in the Wnt pathway is essential for the oscillating expressions of Wnt target genes, but has no influence on Notch target genes.

Lastly, we investigated the regulatory effect of Nkd1 on the crosstalk between the Notch and Wnt pathways. Aki Ishikawa et al. [25] indicated that Nkd1 is a downstream gene of the Wnt pathway, but it is also regulated by Hes7, a downstream gene of the Notch pathway. Nkd1 was expressed in the same phase as Lfng in the middle PSM [25]. Yan et al. [26] and KA Wharton et al. [27] found that Nkd1 inhibits the Wnt pathway by binding with Dsh. From Figure 6 (A) we can see that expression of the Nkd1 gene was oscillating in the same phase as Lfng and Hes7 under normal oscillation conditions. This is in accord with the biological facts. After Wnt signals were knocked out at time point 240 minutes, the expression level of the Nkd1 gene decreased, but still oscillated (see Figure 7 (E)). This suggests that the Nkd1 gene is enhanced by Wnt signals. Wnt signals regulate the expression of the Dll1 gene, which is the ligand of the Notch pathway, so when Wnt signals are removed the Notch pathway target genes will be influenced. In order to see whether or not the regulation of the Nkd1 gene by Wnt signals is through the Notch pathway, we knocked out the Dll1 gene at time point 240 minutes. It was found that the expression level of the Nkd1gene increased rather than decreased (see Figure 7 (F)). This is different from the simulation result of Wnt knockout. So we conclude that the regulation of Nkd1 by Wnt signals is not through the Notch pathway. These observations are supported by the research of Aki Ishikawa et al. [25]. Next, we knocked out the Hes7 gene at time point 120 minutes. It was found that the Nkd1gene was upregulated and ceased to oscillate (see Figure 7 (G)). After the Nkd1 gene was knocked out at time point 120 minutes, the expression levels of Wnt target genes (i.e. amplitudes) increased with a time delay about 60 minutes, whereas the expression levels of the Notch target genes decreased immediately and then began to increased about one period later (see Figure 7 (H)). Therefore, Hes7 is essential for the oscillating expression of Nkd1, and Nkd1 inhibits the Wnt pathway, though there is a time delay. These observations are supported by the research of Aki Ishikawa et al. [25], Yan et al. [26] and KA Wharton et al. [27]. By the mathematical model, we can give a reasonable explanation of these observations. Nkd1 competes with NICD for Dsh. When Nkd1 is knocked out, more NICD binds to Dsh, so that the unbound NICD concentration decreases. As a result, the expression levels of Notch target genes decrease. But after a while, the expression of the Dll1 gene begin to rise owing to the knocking-out of Nkd1, so that the expression levels of Notch target genes rise with it.

In summary, the simulation results demonstrate that the combined model of the Notch and Wnt pathways is capable of simulating the oscillation of the whole network, and can also reproduce wet experimental results. The model has the potential for further use in research on the molecular mechanisms of somitogenesis.

### Parameters sensitivity analysis of the models

The parameter sensitivity analysis is described in the methods section. Here we define a parameter as having significant sensitivity if its sensitivity value is equal to or greater than 1. In simulation experiments, different perturbation levels, low (perturbation 1%), medium (perturbation 10%) and high (perturbation 50%), were used for the parameter sensitivity analysis. All the analysis results are presented in Figures S1 to S12 in Additional file 2. The parameters with significant sensitivity in oscillating periods or amplitudes were picked out and presented in Table S7 to Table S9 in Additional file 2. From the tables we can summarize the characteristics of the isolated pathway models’ sensitivities as follows. (1) The isolated Wnt pathway model is robust to nearly all the parameters in the period and amplitude at all three levels. (2) The periods of target genes in the single Notch pathway model are robust to most of the parameters, and are significantly sensitive to no more than five parameters relevant to the Lfng and Hes7 genes. (3) The target genes in the single Notch pathway model show a diverse spectrum of amplitude sensitivities at the same or different perturbation levels. For the combined model, the periods of the Notch and Wnt target genes are sensitive to the parameters relevant to the Notch pathway, especially the Lfng and Hes7 genes, while they are robust to the parameters relevant to the Wnt pathway. This suggests that the period of somitogenesis is more prone to abnormalities when the Notch pathway is disturbed. This conclusion is in accord with the findings of Leah Herrgen et al. [28]. The amplitudes of the Wnt target genes in the combined model tend to be more sensitive than in the isolated Wnt pathway model.

## Discussion

In this study, we established three mathematical models concerning the Notch and Wnt pathways and their crosstalk during somitogenesis. These models can be used to simulate the oscillating expressions of the target genes of the Notch and/or Wnt pathways and to explore the molecular mechanism of network oscillation. Because the experimental data are limited, this research mainly focuses on the oscillation characteristics of target genes and trends of concentration change of molecules in the pathways, rather than precise experimental results. So the proposed models are qualitative rather than quantitative. For more precise modeling, more experimental data on somitogenesis are still desirable. In addition, it is known that the Notch and Wnt pathways in a single cell are regulated by ligands from adjacent cells in the PSM. In this study, we assumed for simplicity that the pathways in a single cell are regulated by the ligands from that cell. The somitogenesis-related pathways of the cells in the PSM are oscillation-synchronous in somitogenesis, so this assumption has a rational basis. We just modeled the dynamics of the Notch and Wnt pathways in a single cell here. We will consider the communication between cells and establish mathematical models among a group of cells in the PSM in future research.

## Conclusions

Although some models for the Notch and Wnt pathways in somitogenesis have been proposed in previous research, these models are too simple for use in researching the molecular mechanism of somitogenesis. Based on recent literature, we proposed two more complicated models for the isolated Notch and Wnt pathways by improving structure design and the representation of chemical reactions. On this basis, we proposed a new structure of the combined model by considering multiple levels of crosstalk between the two pathways. These models are able to simulate the periodic expressions of the Notch or/and Wnt target genes correctly, and can also reproduce biological experimental results from many different publications. They have the potential for use in exploring the molecular mechanisms of the Notch and Wnt signaling pathways and their crosstalk during somitogenesis. The simulation experiments demonstrate that the extracellular signals of the Notch and Wnt signaling pathways are essential for the oscillating expressions not only of their own but also each other’s target genes. Moreover, the negative feedback loops in the Notch and Wnt pathways play important but distinct roles in maintaining the system oscillation. The parameter sensitivity analysis of the models suggests that compared to the Wnt pathway, the Notch pathway is more sensitive to perturbation, so it is prone to abnormalities in somitogenesis.

## Methods

### The approaches to modeling

All models in this study were established using ODEs. The structure and the equations of the models were created using CellDesigner [29], and were exported as SBML format files. The SBML files were imported into MATLAB and the format converted, so that they can be read by the SBToolBox [30]. In the SBToolBox framework, we accomplished the tasks of parameter learning and parameter sensitivity analysis. Ultimately, the final models were imported into COPASI [31]. All of the model simulations were performed under the COPASI environment. The equations of the models are presented in Additional file 1. The models established using CellDesigner, COPASI and SBToolBox are presented in Additional file 3.

### Parameter learning algorithm

We first assigned each species a random initial value, and then trained the model parameters. In general, the system reached semi-steady oscillation after a few abnormal periods. When the semi-steady oscillation was reached, the values of species at that time point were chosen as their initial values. Some of the parameters in the models were derived from [12], but most of them were estimated by using the parameter learning algorithm. The training set for parameter learning was a set of microarray data derived from [32] available in Additional file 3. The initial values and parameters of the three models are presented in Tables S1 to S6 in Additional file 1.

In this study, a parameter learning algorithm based on particle swarm optimization (PSO) and simulated annealing (SA) was proposed and the following fitness functions were adopted.

(1) $$S=\sum_{i=1}^{N}1-\left|\mathrm{corr}\left(\tilde{V_{i}},V_{i}\right)\right|$$

(2) $$\mathrm{corr}\left(X,Y\right)=\frac{\sum_{j=1}^{M}\left(x_{j}-\bar{x}\right)\left(y_{j}-\bar{y}\right)}{\sqrt{\sum_{j=1}^{M}{\left(x_{j}-\bar{x}\right)}^{2}}\sqrt{\sum_{j=1}^{M}{\left(y_{i}-\bar{y}\right)}^{2}}}$$

Where *N* is the number of target genes; $\tilde{V_{i}}$ is the simulating expression profile of the *i* th target gene; *V*_*i*_ is the real expression profile of the *i* th target gene; *M* is the time point number of the gene expression profile; *x*_*j*_ is the expression value of gene expression profile *X* at time point *j*; $\bar{x}$ is the average expression value of gene expression profile *X* at all time points; *y*_*j*_ is the expression value of gene expression profile *Y* at time point *j*; $\bar{y}$ is the average expression value of gene expression profile *Y* at all time points; *corr* (*X*, *Y*) is the correlation coefficient between gene expression profiles *X* and *Y*. A PSO-embedded SA algorithm was proposed to explore the optimal parameter set. The algorithm pseudocode is presented in Additional file 4. Its MATLAB code is available in Additional file 5.

### Parameter sensitivity analysis

The oscillating periods and amplitudes of the target genes were taken as the outputs of systems for the sensitivity analysis. The formulae for the sensitivity analysis are defined as in equations (3) and (4) [33,34].

(3) $$S_{\tau }=\frac{\partial \tau }{\partial p}\times \frac{p}{\tau }=\frac{\left(\tau_{\mathrm{pert}}-\tau_{\mathrm{norm}}\right)}{\left(p_{\mathrm{pert}}-p_{\mathrm{norm}}\right)}\times \frac{p_{\mathrm{norm}}}{\tau_{\mathrm{norm}}}$$

(4) $$S_{A}=\frac{\partial A}{\partial p}\times \frac{p}{A}=\frac{\left(A_{\mathrm{pert}}-A_{\mathrm{norm}}\right)}{\left(p_{\mathrm{pert}}-p_{\mathrm{norm}}\right)}\times \frac{p_{\mathrm{norm}}}{A_{\mathrm{norm}}}$$

Where *S*_*τ*_ is the period sensitivity; *τ*_*norm*_ is the period of a gene’s oscillation at normal parameter value; *τ*_*pert*_ is the period of a gene’s oscillation after perturbation; *p*_*norm*_ is the normal parameter value; *p*_*pert*_ is the perturbed parameter value; *S*_*A*_ is the amplitude sensitivity; *A*_*norm*_ is the amplitude of a gene’s oscillation at normal parameter value; *A*_*pert*_ is the amplitude of a gene’s oscillation after perturbation.

## Competing interests

The authors declare that they have no competing interests.

## Authors’ contributions

YXH and HYW conceived and designed the research. HYW, YFQ, YZ, LHZ and YWZ performed the research including data collection, testing and analysis. YLB, LGS and ZQM suggested extensions and modifications to the research. YXL supervised the whole research and revised the manuscript critically. All authors read and approved the final manuscript.

## Supplementary Material

Additional file 1 **The ODEs, parameters and initial values of the species.** The ODEs of the models are described at the beginning of the file. The parameters and initial values of the models are in **Tables S1** to S6. Click here for file

Additional file 2 **The results of parameter sensitivity analysis.Figures S1** to S12 present the results of the parameter sensitivity analysis of the three models and **Tables S7** to S9 contain the parameters to which the models are significantly sensitive. Click here for file

Additional file 3 **The models established By CellDesigner, COPASI and SBToolBox.** This file contains three kinds of models established using CellDesigner, COPASI and SBToolBox for MATLAB. Click here for file

Additional file 4 **The pseudocode of the parameter learning algorithm.** This file contains the pseudocode of the parameter learning algorithm proposed in this paper, which is described by a process design language. Click here for file

Additional file 5 **The MATLAB code of the parameter learning algorithm.** This file contains the MATLAB code of the parameter learning algorithm, which runs in the Linux platform. Click here for file
