# Supplementary material for: Mathematical models for the Notch and Wnt signaling pathways and the crosstalk between them during somitogenesis
Source: Theor Biol Med Model. 2013 Apr 20;10:27. doi: 10.1186/1742-4682-10-27 (PMC3648501; doi:10.1186/1742-4682-10-27)
Supplement: Additional file 1 — The ODEs, parameters and initial values of the species. The ODEs of the models are described at the beginning of the file. The parameters and initial values of the models are in Tables S1 to S6. [file 1742-4682-10-27-S1.pdf]

The ODEs modelling the single Notch pathway

The single Notch pathway model consists of 12 ODEs from Eq 1.1 to Eq 1.12. We described every equation as follows.

(1) The Dll1 is the ligand of Notch pathway. We assumed that it is synthesized at a constant rate and its degradation obeys Michaelis-Menten kinetics.

$$\frac{d[Dll1]}{dt} = v_{sDll1} - \mathbf{deg}(Dll1) \qquad \text{Eq 1.1}$$

Where [Dll1] represents the Dll1 concentration. Without specific description, '[X]' represents the concentration of species 'X' in this paper. v_{sDll1} represents the synthesis rate of the Dll1 (see **Table S1**). $\mathbf{deg}(Dll1)$ represents the degradation of the Dll1. The definition of function '**deg**' can refer to Eq 1.13.

(2) The Notch is the receptor of the Notch pathway. We also assumed that it is synthesized with a constant rate and its degradation obeys Michaelis-Menten kinetics. Under the conditions of the activation of Dll1 and the inhibition of Lfng, NICD is decomposed from the Notch. The activation of the Dll1 was modeled using a Hill equation with Hill coefficient 1 because we supposed that the Dll1 protein catalyzes the Notch at only one site and the inhibition of Lfng was modeled using Hill equation with Hill coefficient 'n' because the Lfng inhibits the Notch at multiple sites (n=2 in this paper). The meaning of the parameters in Eq 1.2 can refer to **Table S1.**

$$\frac{d[Notch]}{dt} = v_{sNotch} - k_c[Notch]\left(\frac{[Dll1]}{K_{DLL1}+[Dll1]}\right)\left(\frac{K_{ILfng}{}^{n}}{K_{ILfng}{}^{n}+[Lfng]^{n}}\right) - \mathbf{deg}(Notch) \qquad \text{Eq 1.2}$$

(3) The NICD protein is the major transcription factor of the Notch pathway. The NICD protein in the cytoplasm is decomposed from the Notch receptor under the activation of Dll1 and the inhibition of Lfng (reaction equation can refer to Eq 1.2). In addition, it can shuttle between cytoplasm and nucleus, and this are modeled using two mass action equations because the shuttling rates are in proportion to the concentrations of the reactants in the compartment. Its degradation is modeled by a

Michaelis-Menten equation. The equation of the NICD in the cytoplasm is shown in Eq 1.3. The meaning of the parameters in Eq 1.3 can refer to **Table S1.**

$$\frac{d\left[NICD_c\right]}{dt}=k_c\left[Notch\right]\left(\frac{\left[DLL1\right]}{K_{DLL1}+\left[DLL1\right]}\right)\left(\frac{K_{ILfng}{}^{n}}{K_{ILfng}{}^{n}+\left[Lfng\right]^{n}}\right) + EX_{NICDn}\left[NICD_n\right]-IM_{NICDc}\left[NICD_c\right]-\mathbf{deg}\left(NICD_c\right) \quad \text{Eq 1.3}$$

Where, the subscript 'c' represents the species located in the cytoplasm and the subscript 'n' represents the species in the nucleus. So are the following equations.

(4) The Lfng protein is translated from the Lfng mRNA in the cytoplasm, and this is modeled using a mass action equation because we supposed that the synthesis rate of the protein is in proportion to the concentration of the mRNA. Its degradation is modeled using a Michaelis-Menten equation. The equation of the Lfng protein is shown in Eq 1.4. The meaning of parameters in Eq 1.4 can refer to **Table S1.**

$$\frac{d\left[Lfng\right]}{dt}=k_{mLfng}\left[mLfng_c\right]-\mathbf{deg}\left(Lfng\right) \quad \text{Eq 1.4}$$

(5) The Lfng mRNA in cytoplasm is transported from nucleus, and this is modeled using a mass action equation because the transportation rate is in proportion to the concentration of the Lfng mRNA in cytoplasm. Its degradation is modeled using a Michaelis-Menten equation. The equation of the Lfng mRNA is shown in Eq 1.5. The meaning of parameters in Eq 1.5 can refer to **Table S1.**

$$\frac{d\left[mLfng_c\right]}{dt}=EX_{mLfngn}\left[mLfng_n\right]-\mathbf{deg}\left(mLfng_c\right) \quad \text{Eq 1.5}$$

Where, $mLfng$ represents the Lfng mRNA. Without specific description, prefix 'm' in a species name represents the mRNA of this species in this paper.

(6) The Hes7 protein in cytoplasm is translated from the Hes7 mRNA in cytoplasm, and this is modeld using a mass action equation because the translation rate is in proportion to the concentration of the Hes7 mRNA in the cytoplasm. Its degradation is modeled using a Michaelis-Menten equation. In addition, the Hes7 protein can shuttle between cytoplasm and nucleus, and this is modeled using a mass action equation because the shuttling rate is in proportion to the concentration of the reactants in the

compartment. The equation of the Hes7 protein in the cytoplasm is shown in Eq 1.6. The meaning of parameters in Eq 1.6 can refer to **Table S1.**

$$\frac{d\left[Hes7_c\right]}{dt}=k_{mHes7}\left[mHes7_c\right]+EX_{Hes7n}\left[Hes7_n\right] \\ -IM_{Hes7c}\left[Hes7_c\right]-\mathbf{deg}\left(Hes7_c\right) \qquad \text{Eq 1.6}$$

(7) The Hes7 mRNA in cytoplasm is transported from nucleus, and this is modeled using a mass action equation because the transportation rate is in proportion to the concentration of the Hes7 mRNA in the cytoplasm. Its degradation is modeled using a Michaelis-Menten equation. The equation of the Hes7 mRNA is shown in Eq 1.7. The meaning of parameters in Eq 1.7 can refer to **Table S1**.

$$\frac{d\left[mHes7_c\right]}{dt}=EX_{mHes7n}\left[mHes7_n\right]-\mathbf{deg}\left(mHes7_c\right) \qquad \text{Eq 1.7}$$

(8) The NICD protein in nucleus is transported from cytoplasm and it can also be transported into cytoplasm from nucleus. The shuttle of the NICD between the cytoplasm and the nucleus is modeled using a mass action equation because the shuttling rate is in proportion to the concentration of the reactants in the compartment. It can reversibly bind to the RBP-j protein in the nucleus and this is modeled using a mass action equation because the binding and decomposed rates are in proportion to the concentration of the reactants. In addition, the NICD in the nucleus is also degraded by a Michaelis-Menten equation. The equation of the NICD protein in the nucleus is shown in Eq 1.8. The meaning of parameters in Eq 1.8 can refer to **Table S1.**

$$\frac{d\left[NICD_n\right]}{dt}=IM_{NICDc}\left[NICD_c\right]+d_{N|R}\left[N\,|\,R\right]-EX_{NICDn}\left[NICD_n\right] \\ -a_{N|R}\left[NICD_n\right]\left[RBP\text{-}j\right]-\mathbf{deg}\left(NICD_n\right) \qquad \text{Eq 1.8}$$

Where, $N\,|\,R$ represents the complex of the NICD and the RBP-j.

(9) The complex of NICD and RBP-j is a transcription activator of the Notch pathway. It is formed by the reversible binding of the NICD protein to the RBP-j protein. The reaction is modeled using a mass action equation because their binding and decomposed rates are in proportion to the concentration of the reactants. The equation

of the complex of NICD and RBP-j in the nucleus is shown in Eq 1.9. The meaning of parameters in Eq 1.9 can refer to **Table S1.**

$$\frac{d[N|R]}{dt} = a_{N|R}[NICD_n][RBP\text{-}j] - d_{N|R}[N|R] \qquad \text{Eq 1.9}$$

(10) The Hes7 protein in nucleus is a major repressor of the Notch pathway. It shuttles between the cytoplasm and the nucleus, this are modeled using two mass action equations because the shuttling rates are in proportion to the concentrations of the reactants in the compartment. In addition, its degradation in the nucleus is modeled using a Michaelis-Menten equation. The equation of the Hes7 protein in the nucleus is shown in Eq 1.10. The meaning of parameters in Eq 1.10 can refer to **Table S1.**

$$\frac{d[Hes7_n]}{dt} = IM_{Hes7c}[Hes7_c] - EX_{Hes7n}[Hes7_n] - \mathbf{deg}(Hes7_n) \qquad \text{Eq 1.10}$$

(11) The Lfng mRNA in nucleus is transcribed from the Lfng gene under conditions of the activation of the NICD-RBP-j complex and the repression of the Hes7 protein. The activation of the NICD-RBP-j complex to the Lfng is modeled using a Hill equation with a positive Hill coefficient and the repression of the Hes7 protein to Lfng is modeled using a Hill equation with a negative Hill coefficient. In addition, the Lfng gene is expressed with a constant rate under condition of no transcription factors due to transcriptional leakage. After transcription has terminated, the Lfng mRNA is transported into the cytoplasm, and this is modeled using a mass action equation because the transportation rate is in proportion to the concentration of the Lfng mRNA in the nucleus. The equation of the Lfng mRNA in the nucleus is shown in Eq 1.11. The meaning of parameters in Eq 1.11 can refer to **Table S1.**

$$\begin{aligned}\frac{d[mLfng_n]}{dt} &= v_{mLfng} + V_{MsmLfng}\left(\frac{[N|R]^{h1}}{K_{smLfng}{}^{h1} + [N|R]^{h1}}\right) \times \mathbf{Inh}(Hes7, mLfng_n) \\ &\quad - EX_{mLfngn}[mLfng_n]\end{aligned} \qquad \text{Eq 1.11}$$

Where $\mathbf{Inh}(Hes7, mLfng_n)$ represents the repression of the Hes7 protein to the Lfng gene. The definition of function '**Inh**' can refer to Eq 1.14.

(12) The Hes7 mRNA is transcribed from the Hes7 gene under conditions of the

activation of the NICD-RBP-j complex and the repression of the Hes7 protein. The activation of the NICD-RBP-j complex to the Hes7 gene is modeled using a Hill equation with a positive Hill coefficient and the repression of the Hes7 protein to the Hes7 gene is modeled using a Hill equation with a negative Hill coefficient. The Hes7 gene is expressed with a constant rate under condition of no transcription factors due to transcriptional leakage. After transcription has terminated, the Hes7 mRNA is transported into the cytoplasm, this is modeled using a mass action equation because the transportation rate is in proportion to the concentration of the Hes7 mRNA in the nucleus. The equation of the Hes7 mRNA in the nucleus is shown in Eq 1.12. The meaning of parameters in Eq 1.12 can refer to **Table S1**.

$$\frac{d[mHes7_n]}{dt} = v_{mHes7} + V_{MsmHes7}\left(\frac{[N|R]^{h2}}{K_{smHes7}{}^{h2} + [N|R]^{h2}}\right) \times \mathbf{Inh}(Hes7, mHes7_n) - EX_{mHes7n}[mHes7_n] \quad \text{Eq 1.12}$$

$$\mathbf{deg}(A) = V_{MdA}\left(\frac{[A]}{K_{dA} + [A]}\right) \quad \text{Eq 1.13}$$

$$\mathbf{Inh}(X,Y) = \frac{K_{XIY}{}^{hXIY}}{K_{XIY}{}^{hXIY} + [X]^{hXIY}} \quad \text{Eq 1.14}$$

In this model we assumed the total concentration of the RBP-j protein is constant. The RBP-j protein can only exist in two states, dissociative from or forming a complex with NICD, so the concentration of dissociative RBP-j is presented in Eq 1.15. It is a boundary condition of this model.

$$[RBP\text{-}j] = RBPj_t - [N|R] \quad \text{Eq 1.15}$$

The initial values in the model are shown in the **Table S2**.

The ODEs modelling the single Wnt pathway

The single Wnt pathway model consists of 13 ODEs. They are Eq 2.1 to Eq 2.13 and we described them as follows.

(1) In the upstream of the Wnt pathway, the Dsh ligand is an important medium of Wnt signals. In this model, we supposed that the concentration of the Dsh ligand is

constant so there are no synthesis and degradation reactions of the Dsh ligand. Here we supposed the Dsh ligand exists in two states, active and inactive, and only the active Dsh can activate Wnt signals. In this model the Dsh ligand can reversibly be activated by the Wnt receptor. The activation reaction of the Dsh ligand is modeled using a Michaelis-Menten equation because we supposed that the activation obeys an enzymatic reaction law and the activated effect of Wnt to Dsh is modeled using a Hill equation with Hill coefficient 1 because we supposed Wnt binds to Dsh at only one site. In addition, the active Dsh can bind to the Axin2 protein to form a complex and this complex can be degraded, so that the Dsh in the complex can revert to the active Dsh. This reaction is modeled using a mass action equation because the reaction rate is in proportion to the concentration of the reactants. In summary, the equation of the active Dsh is shown in Eq 2.1. The concentration of the inactive Dsh is shown in Eq 2.14 and it is a boundary condition of the model.

$$\begin{aligned}\frac{d[Dsh_{act}]}{dt} = & V_{MaDsh}\left(\frac{[Wnt]}{K_{aWnt}+[Wnt]}\right)\left(\frac{[Dsh_{inact}]}{K_{aDsh}+[Dsh_{inact}]}\right)+k_{D|A}[D|A] \\ & -V_{MinaDsh}\left(\frac{[Dsh_{act}]}{K_{inaDsh}+[Dsh_{act}]}\right)-a_{D|A}[Axin2][Dsh_{act}]\end{aligned} \qquad \text{Eq 2.1}$$

Where 'Wnt' represents the complex of Wnt and Frizzeld, $D|A$ represents the complex of the Dsh and the Axin2 protein. Other parameters of the equation can be found in the **Table S3**.

(2) The complex of the active Dsh and the Axin2 protein is formed by the binding of the active Dsh to the Axin2 protein and when the complex is degraded, the Dsh can be reverted to the active Dsh. During this process, the Axin2 protein is degraded by the active Dsh. This reaction is modeled using a mass action equation because the reaction rate is in proportion to the concentration of the reactants. The equation of the Dsh-Axin2 complex is shown in Eq 2.2. The meaning of parameters in Eq 2.2 can refer to **Table S3**.

$$\frac{d[D|A]}{dt} = a_{D|A}[Axin2][Dsh_{act}] - k_{D|A}[D|A] \qquad \text{Eq 2.2}$$

(3) The complex of the GSK3 and the Axin2 is the important component of the degradation complex in which the β-catenin is quickly phosphorylated and degraded. It is reversibly formed by the binding of the GSK3 protein and the Axin2 protein. This reaction is modeled using a mass action equation because the reaction rate is in proportion to the concentration of the reactants. The equation of the GSK3-Axin2 complex is shown in Eq 2.3. The meaning of parameters in Eq 2.3 can refer to **Table S3**.

$$\frac{d[G|A]}{dt} = a_{G|A}[GSK3][Axin2] - d_{G|A}[G|A] \qquad \text{Eq 2.3}$$

Where $G|A$ represents the complex of the GSK3 protein and the Axin2 protein.

(4) The Axin2 protein is translated from the Axin2 mRNA, and this is modeled using a mass action equation because the translation rate is in proportion to the concentration of the Axin2 mRNA in the cytoplasm. In addition, it can reversibly bind to the GSK3 protein and the active Dsh, and this is modeled using two mass action equations because the reaction rates are in proportion to the concentration of the reactants. The equation of the Axin2 protein is shown in Eq 2.4. The meaning of parameters in Eq 2.4 can refer to **Table S3**.

$$\begin{aligned}\frac{d[Axin2]}{dt} &= k_{mAxin2}[mAxin2_c] + d_{G|A}[G|A] - a_{G|A}[GSK3][Axin2] \\ &\quad - a_{D|A}[Dsh_{act}][Axin2]\end{aligned} \qquad \text{Eq 2.4}$$

(5) The β-catenin protein is the major transcription factor of the Wnt pathway. In this model we supposed that it is synthesized with a constant rate. Moreover, it exists in two states in the cytoplasm, unphosphorylated and phosphorylated. The unphosphorylated β-catenin protein can be reversibly phosphorylated by the GSK3-Axin2 complex, and this is modeled using a Michaelis-Menten equation because we supposed that the activation obeys an enzymatic reaction law and the activation rate of the GSK3-Axin2 complex to the phosphorylation of the β-catenin protein is in proportion to the percentage of the GSK3 protein in the GSK3-Axin2 complex to the total concentration of the GSK3 protein.

In addition, the unphosphorylated β-catenin protein can shuttle between the cytoplasm and the nucleus, and this is modeled using a mass action equation because the shuttling rate is in proportion to the concentration of the reactants. The equation of the unphosphorylated β-catenin protein is shown in Eq 2.5. The meaning of parameters in Eq 2.5 can refer to **Table S3**.

$$\frac{d[\beta\text{-}cat]}{dt} = v_{\beta cat} + EX_{\beta catn}[\beta\text{-}cat_n] + V_{Md\beta catp}\left(\frac{[\beta\text{-}cat_p]}{K_{d\beta catp}+[\beta\text{-}cat_p]}\right) - IM_{\beta cat}[\beta\text{-}cat] - V_{Ma\beta catp}\left(\frac{[G|A]}{GSK3_t}\right)\left(\frac{[\beta\text{-}cat]}{K_{a\beta catp}+[\beta\text{-}cat]}\right) \quad \text{Eq 2.5}$$

Where, $\beta\text{-}cat$ represents the unphosphorylated β-catenin protein and $\beta\text{-}cat_p$ represents the phosphorylated β-catenin protein.

(6) The phosphorylated β-catenin protein comes from reversible phosphorylation of the unphosphorylated β-catenin protein and it can be degraded quickly with a first-order action law. Its equation is shown in Eq 2.6. The meaning of parameters in Eq 2.6 can refer to **Table S3**.

$$\frac{d[\beta\text{-}cat_p]}{dt} = V_{Ma\beta catp}\left(\frac{[G|A]}{GSK3_t}\right)\left(\frac{[\beta\text{-}cat]}{K_{a\beta catp}+[\beta\text{-}cat]}\right) - V_{Md\beta catp}\left(\frac{[\beta\text{-}cat_p]}{K_{d\beta catp}+[\beta\text{-}cat_p]}\right) - k_{d1}[\beta\text{-}cat_p] \quad \text{Eq 2.6}$$

(7) The Dll1 protein is translated from the Dll1 mRNA, and this is modeled using a mass action equation because we supposed that the synthesis rate of the Dll1 protein is in proportion to the concentration of the Dll1 mRNA. The degradation of the Dll1 protein is modeled using a Michaelis-Menten equation. The equation of the Dll1 protein is shown in Eq 2.7. The meaning of parameters in Eq 2.7 can refer to **Table S3.**

$$\frac{d[Dll1]}{dt} = k_{mDll1}[mDll1_c] - \mathbf{deg}(Dll1) \quad \text{Eq 2.7}$$

(8) The Axin2 mRNA in the cytoplasm is transported from the nucleus, and this is

modeled using a mass action equation because the transportation rate is in proportion to the concentration of the Axin2 mRNA in the cytoplasm. Its degradation is modeled using a Michaelis-Menten equation. The equation of the Axin2 mRNA is shown in Eq 2.8. The meaning of parameters in Eq 2.8 can refer to **Table S3.**

$$\frac{d[mAxin2_c]}{dt} = EX_{mAxin2n}[mAxin2_n] - \mathbf{deg}(mAxin2_c) \quad \text{Eq 2.8}$$

(9) The Dll1 mRNA in the cytoplasm is transported from the nucleus, and this is modeled using a mass action equation because the transportation rate is in proportion to the concentration of the Dll1 mRNA in the cytoplasm. Its degradation is modeled using a Michaelis-Menten equation. The equation of the Dll1 mRNA is shown in Eq 2.9. The meaning of parameters in Eq 2.9 can refer to **Table S3.**

$$\frac{d[mDll1_c]}{dt} = EX_{mDll1n}[mDll1_n] - \mathbf{deg}(mDll1_c) \quad \text{Eq 2.9}$$

(10) The β-catenin protein in nucleus is reversibly transported from the cytoplasm, and this is modeled using a mass action equation because the transportation rate is in proportion to the concentration of the reactants. In addition, it can reversibly bind to the Lef1 protein to form a complex in the nucleus, and this is modeled using a mass action equation. The equation of the β-catenin protein in the nucleus is shown in Eq 2.10. The meaning of parameters in Eq 2.10 can refer to **Table S3.**

$$\begin{aligned}\frac{d[\beta\text{-}cat_n]}{dt} = {} & IM_{\beta cat}[\beta\text{-}cat] + d_{\beta|L}[\beta|L] \\ & - a_{\beta|L}[\beta\text{-}cat_n][Lef1_n] - EX_{\beta catn}[\beta\text{-}cat_n]\end{aligned} \quad \text{Eq 2.10}$$

Where $\beta|L$ represents the complex of β-catenin and Lef1 which regulates the target genes' expression in the Wnt pathway.

(11) The β-catenin-Lef1 complex is a transcription activator of the Wnt pathway and is formed by the reversible binding of β-catenin to the Lef1 protein, and this is modeled using a mass action equation. Its equation is shown in Eq 2.11. The meaning of parameters in Eq 2.11 can refer to **Table S3.**

$$\frac{d[\beta|L]}{dt} = a_{\beta|L}[\beta\text{-}cat_n][Lef1_n] - d_{\beta|L}[\beta|L] \quad \text{Eq 2.11}$$

(12) The Axin2 mRNA in the nucleus is transcribed from the Axin2 gene under the regulation of the β-catenin-Lef1 complex, and this is modeled using a Hill equation with a Hill coefficient 'h3'. In addition, the Axin2 gene is expressed with a constant rate under condition of no transcription factors due to transcriptional leakage. After transcription has terminated, the Axin2 mRNA is transported from the nucleus. This is modeled using a mass action equation. Its equation is shown in Eq 2.12. The meaning of parameters in Eq 2.12 can refer to **Table S3**.

$$\frac{d\left[mAxin2_n\right]}{dt} = v_{mAxin2} + V_{MsmAxin2}\left(\frac{\left[\beta \mid L\right]^{h3}}{K_{smAxin2}{}^{h3} + \left[\beta \mid L\right]^{h3}}\right) - EX_{mAxin2n}\left[mAxin2_n\right] \quad \text{Eq 2.12}$$

(13) The Dll1 mRNA in the nucleus is transcribed from the Dll1 gene under the regulation of the β-catenin-Lef1 complex, and this is modeled using a Hill equation. In addition, the Dll1 gene is expressed with a constant rate under condition of no transcription factors due to transcriptional leakage. After transcription has terminated, the Dll1 mRNA is transported from the nucleus. This is modeled using a mass action equation. Its equation is shown in Eq 2.13. The meaning of parameters in Eq 2.13 can refer to **Table S3**.

$$\frac{d\left[mDll1_n\right]}{dt} = v_{mDll1} + V_{MsmDll1}\left(\frac{\left[\beta \mid L\right]^{h4}}{K_{smDll1}{}^{h4} + \left[\beta \mid L\right]^{h4}}\right) - EX_{mDll1n}\left[mDll1_n\right] \quad \text{Eq 2.13}$$

In addition, in this model we assume the total concentration of Dsh, GSK3 and Lef1 is constant. The Dsh protein can exist in 3 states, active, inactive, and forming complex with Axin2, so the concentration of inactive Dsh is presented in Eq 2.14. The GSK3 protein can only exist in 2 states, dissociative from and forming complex with Axin2, so the concentration of dissociative GSK3 is presented in Eq 2.15. The Lef1 protein can only exist in 2 states, dissociative from and forming complex with β-catenin, so the concentration of dissociative Lef1 is presented in equation Eq 2.16. The initial values in this model are in the **Table S4**.

$$\left[Dsh_{inact}\right] = Dsh_t - \left[Dsh_{act}\right] - \left[D \mid A\right] \quad \text{Eq 2.14}$$

$$\left[GSK3\right] = GSK3_t - \left[G \mid A\right] \quad \text{Eq 2.15}$$

$$[Lef1] = Lef1_t - [\beta | L] \qquad \text{Eq 2.16}$$

The ODEs modelling the crosstalk model between the Notch and Wnt pathways

The crosstalk model comes from the combination of the single Notch pathway model and the single Wnt pathway model by adding some species and chemical reactions to the previous two models. Next we described the added and modified ODEs in the crosstalk model.

(1) The Dll1 protein is included in both the single Notch pathway model and the single Wnt pathway model. There are two different ODEs of the Dll1 protein in the two models. Because the Dll1 protein is translated from the Dll1 mRNA, in the crosstalk model, the Eq 2.7 in the single Wnt pathway model is preserved and the Eq 1.1 in the single Notch pathway model is removed from the crosstalk model.

(2) To model the mutual inhibition of the Notch pathway and the Wnt pathway through the NICD protein and the Dsh protein, we added a new reaction which is the reversible binding of the NICD protein to the active Dsh protein in the crosstalk model. We model this reaction using a mass action equation because the reaction rates are in proportion to the reactants. The equation of the NICD-Dsh complex is shown in Eq 3.1. The meaning of parameters in Eq 3.1 can refer to **Table S5.**

$$\frac{d[N|D]}{dt} = a_{N|D}[NICD_c][Dsh_{act}] - d_{N|D}[N|D] \qquad \text{Eq 3.1}$$

Where, $N|D$ represents the complex of the NICD protein and the active Dsh protein.

(3) Because the NICD protein in the cytoplasm can bind to the active Dsh protein in the crosstalk model, the ODE of the NICD protein in the cytoplasm (Eq 1.3) should be modified. The equation of the NICD protein in the cytoplasm in the crosstalk model is shown in Eq 3.2. The meaning of parameters in Eq 3.2 can refer to **Table S5.**

$$\begin{aligned}\frac{d[NICD_c]}{dt} = {} & k_c[Notch]\left(\frac{[Dll1]}{K_{Dll1}+[Dll1]}\right)\left(\frac{K_{ILfng}{}^n}{K_{ILfng}{}^n+[Lfng]^n}\right) \\ & + d_{N|D}[N|D] + EX_{NICDn}[NICD_n] - a_{N|D}[NICD_c][Dsh_{act}] \\ & - IM_{NICDc}[NICD_c] - \mathbf{deg}(NICD_c)\end{aligned} \qquad \text{Eq 3.2}$$

(4) The Nkd1 gene is a new target gene in the crosstalk model. It is expressed under the activation of the β-catenin-Lef1 complex and the repression of the Hes7 protein. We model the activation of the β-catenin-Lef1 complex to Nkd1 using a Hill equation with a positive Hill coefficient and the repression of the Hes7 protein to Nkd1 using a Hill equation with a negative Hill coefficient. In addition, the Nkd1 gene is expressed with a constant rate under condition of no transcription factors due to transcriptional leakage. After transcription has terminated, the Nkd1 mRNA is transported from the nucleus, and this is modeled using a mass action equation. The equation of the Nkd1 mRNA in the nucleus in the crosstalk model is shown in Eq 3.3. The meaning of parameters in Eq 3.3 can refer to **Table S5**.

$$\frac{d\left[mNkd1_n\right]}{dt} = v_{mNkd1} + V_{MsmNkd1}\left(\frac{\left[\beta\,|\,L\right]^{h3}}{K_{smNkd1}{}^{h3} + \left[\beta\,|\,L\right]^{h3}}\right) \times \mathbf{Inh}\left(Hes7, mNkd1_n\right) - EX_{mNkd1n}\left[mNkd1_n\right] \qquad \text{Eq 3.3}$$

(5) The Nkd1 mRNA in the cytoplasm is transported from the nucleus, and this is modelled using a mass action equation because the transportation rate is in proportion to the concentration of the Nkd1 mRNA in the nucleus. Its degradation is modelled using a Michaelis-Menten equation. The equation of the Nkd1 mRNA in the cytoplasm is shown in Eq 3.4. The meaning of parameters in Eq 3.4 can refer to **Table S5**.

$$\frac{d\left[mNkd1_c\right]}{dt} = EX_{mNkd1n}\left[mNkd1_n\right] - \mathbf{deg}\left(mNkd1_c\right) \qquad \text{Eq 3.4}$$

(6) The Nkd1 protein is translated from the Nkd1 mRNA, and this is modeled using a mass action equation because we supposed that the translation rate is in proportion to the concentration of the Nkd1 mRNA in the cytoplasm. Moreover, it can reversibly bind to the active Dsh protein. This is modeled using a mass action equation. Its degradation is modeled using a Michaelis-Menten equation. The equation of the Nkd1 protein is shown in Eq 3.5. The meaning of parameters in Eq 3.5 can refer to **Table S5**.

$$\frac{d[Nkd1]}{dt} = k_{mNkd1}[mNkd1_c] + d_{K|D}[K|D] - a_{K|D}[Nkd1][Dsh_{act}] - \mathbf{deg}(Nkd1) \qquad \text{Eq 3.5}$$

Where $K|D$ represents the Nkd1-Dsh complex.

(7) The Nkd1-Dsh complex is reversibly formed by the binding of the Nkd1 protein to the active Dsh protein, and this is modelled using a mass action equation. Its equation is shown in Eq 3.6. The meaning of parameters in Eq 3.6 can refer to **Table S5**.

$$\frac{d[K|D]}{dt} = a_{K|D}[Nkd1][Dsh_{act}] - d_{K|D}[K|D] \qquad \text{Eq 3.6}$$

(8) Because the NICD protein and the Nkd1 protein can both reversibly bind to the active Dsh protein in the crosstalk model, thereby the ODE of the active Dsh protein in the single Wnt pathway model (Eq 2.1) should be modified. The equation of the active Dsh protein in the crosstalk model is shown in Eq 3.7. The meaning of parameters in Eq 3.7 can refer to **Table S5**.

$$\begin{aligned}\frac{d[Dsh_{act}]}{dt} = {} & V_{MaDsh}\left(\frac{[Wnt]}{K_{aWnt}+[Wnt]}\right)\left(\frac{[Dsh_{inact}]}{K_{aDsh}+[Dsh_{inact}]}\right) \\ & + k_{D|A}[D|A] + d_{N|D}[N|D] + d_{K|D}[K|D] \\ & - a_{D|A}[Axin2][Dsh_{act}] - a_{N|D}[NICD_c][Dsh_{act}] \\ & - a_{K|D}[Nkd1][Dsh_{act}] - V_{MinaDsh}\left(\frac{[Dsh_{act}]}{K_{inaDsh}+[Dsh_{act}]}\right)\end{aligned} \qquad \text{Eq 3.7}$$

(9) In the crosstalk model the active Dsh can also be included in the NICD-Dsh complex and the Nkd1-Dsh complex, so the concentration of the inactive Dsh in the single Wnt pathway model (Eq 2.14) is changed into Eq 3.8.

$$[Dsh_{inact}] = Dsh_t - [Dsh_{act}] - [D|A] - [N|D] - [K|D] \qquad \text{Eq 3.8}$$

The initial values of the crosstalk model are shown in the **Table S6**.

Table S1. Parameters of the single Notch pathway Model

**Name**	**Description**	**Value**
$RBPj_t$	Total RBP-j concentration	0.35
v_{sDll1}	Dll1 synthesis rate	0.2
v_{sNotch}	Notch synthesis rate	0.069
k_c	Apparent first-order rate constant for Notch cleavage into NICD	1.035
K_{Dll1}	Michaelis constant for Notch cleavage into NICD by Dll1	1
K_{ILfng}	Constant of inhibition by Lfng of Notch cleavage into NICD	0.15
n	Hill coefficient of inhibition by Lfng of Notch cleavage into NICD	2
EX_{NICDn}	Apparent first-order rate constant for NICD exit from nucleus	0.03
IM_{NICDc}	Apparent first-order rate constant for NICD entry into nucleus	0.03
k_{mLfng}	Apparent first-order rate constant for synthesis of Lfng protein	0.09
EX_{mLfngn}	Apparent first-order rate constant for Lfng mRNA exit from the nucleus	0.5
k_{mHes7}	Apparent first-order rate constant for synthesis of Hes7 protein	0.05
IM_{Hes7c}	First-order rate constant for Hes7 protein entry into nucleus	0.01
EX_{Hes7n}	Apparent first-order rate constant for Hes7 protein exit from the nucleus	0.01
EX_{mHes7n}	Apparent first-order rate constant for Hes7 mRNA exit from the nucleus	1.5
$d_{N\|R}$	Bimolecular rate constant for degradation of the complex of NICD and RBP-j	1
$a_{N\|R}$	Bimolecular rate constant for binding of NICD to RBP-j	8
v_{mLfng}	Basal rate of transcription of the Lfng gene	0.03
$V_{MsmLfng}$	Maximum rate of transcription of Lfng gene induced by the complex of NICD and RBP-j in nucleus	0.9

K_{smLfng}	Threshold constant for induction by the complex of NICD and RBP-j in nucleus of Lfng transcription		0.05
$h1$	Hill coefficient of transcription of Lfng induced by the complex of NICD and RBP-j		2
v_{mHes7}	Basal rate of transcription of the Hes7 gene		0.01
$V_{MsmHes7}$	Maximum rate of transcription of Hes7 gene induced by the complex of NICD and RBP-j in nucleus		1.24
K_{smHes7}	Threshold constant for induction by the complex of NICD and RBP-j in nucleus of Hes7 transcription		0.041
$h2$	Hill coefficient of transcription of Hes7 induced by the complex of NICD and RBP-j		2
V_{MdA}	Maximum rate of degradation of A	Dll1	0.385
		Notch	0.846
		$NICD_c$	0.01
		Lfng	0.117
		$mLfng_c$	0.7
		$Hes7_c$	0.1
		$mHes7_c$	0.635
		$NICD_n$	0.003
		$Hes7_n$	0.1
K_{dA}	Michaelis constant for degradation of X	Dll1	1.584
		Notch	1.4
		$NICD_c$	0.001
		Lfng	0.37
		$mLfng_c$	0.768
		$Hes7_c$	0.1
		$mHes7_c$	1
		$NICD_n$	0.001
		$Hes7_n$	0.1

K_{XIY}	Constant of inhibition by X of Y synthesis	Hes7	$mLfng_n$	0.2
		Hes7	$mHes7_n$	0.2
$hXIY$	Hill coefficient of inhibition by X of Y synthesis	Hes7	$mLfng_n$	2
		Hes7	$mHes7_n$	2

First order rate constants are in units of min^{-1} and second order rate constants are expressed in $nM^{-1}min^{-1}$. Michaelis constants and total concentration of species are in nM. Hill coefficients are no units.

The parameter values noted by green font color come from the researching results of Albert Goldbeter at el. [1]. The parameter values noted by black font color are derived from the parameter learning algorithm.

Table S2. Initial values of the single Notch pathway model

**Name**	**Description**	**Value (nM)**
Dll1	The Dll1 protein	1.71
Notch	The Notch protein	0.12
$NICD_c$	The NICD in the cytoplasm	0
Lfng	The Lfng protein	2.72
$Hes7_c$	The Hes7 protein in the cytoplasm	10.66
$mLfng_c$	The Lfng mRNA in the cytoplasm	0.043
$mHes7_c$	The Hes7 mRNA in the cytoplasm	0.042
RBP-j	The RBP-j protein	0.324
$NICD_n$	The NICD in the nucleus	0.009
N|R	The complex of NICD and RBP-j	0.026
$Hes7_n$	The Hes7 protein in the nucleus	1.24
$mLfng_n$	The Lfng mRNA in the nucleus	0.073
$mHes7_n$	The Hes7 mRNA in the nucleus	0.013

Table S3. Parameters of the single Wnt pathway model

Name	Description	Value
Wnt_t	Wnt protein concentration	1.814
Dsh_t	Total Dsh concentration	3.33
$GSK3_t$	Total GSK3 concentration	4.025
$Lef1_t$	Total Lef1 concentration	2.7
V_{MaDsh}	Maximum rate of activation of Dsh by Wnt	5
K_{aWnt}	Michaelis constant for activation of Dsh by Wnt	1.5
K_{aDsh}	Michaelis constant for activation of Dsh	0.95
$V_{MinaDsh}$	Maximum rate of inactivation of Dsh	1
K_{inaDsh}	Michaelis constant for inactivation of Dsh	0.647
$a_{D\|A}$	Bimolecular rate constant for binding of Axin2 to Dsh	10
$k_{D\|A}$	Apparent first-order rate constant for degradation of complex of Dsh and Axin2	0.05
$a_{G\|A}$	Bimolecular rate constant for binding of GSK3 to Axin2	2.7
$d_{G\|A}$	Bimolecular rate constant for degradation of the complex of GSK3 and Axin2	0.1
k_{mAxin2}	Apparent first-order rate constant for synthesis of Axin2 protein	0.03
$V_{Ma\beta catp}$	Maximum rate for phosphorylation of β–catenin	7.62
$K_{a\beta catp}$	Michaelis constant for phosphorylation of β–catenin	0.28
$V_{Md\beta catp}$	Maximum rate for dephosphorylation of β–catenin	1.5
$K_{d\beta catp}$	Michaelis constant for dephosphorylation of β–catenin	0.03
k_{d1}	Apparent first-order rate constant for degradation of phosphorylated β-catenin	10.593
$v_{\beta cat}$	Maximum rate of β–catenin synthesis	0.06

$EX_{\beta catn}$	Apparent first-order rate constant for β–catenin exit from the nucleus		2.25
$IM_{\beta cat}$	Apparent first-order rate constant for β–catenin entry into the nucleus		1.05
k_{mDll1}	Apparent first-order rate constant for synthesis of DLL1 protein		0.01
EX_{mAxin2}	Apparent first-order rate constant for Axin2 mRNA exit from the nucleus		2
EX_{mDll1n}	Apparent first-order rate constant for Dll1 mRNA exit from the nucleus		1.725
$d_{\beta\|L}$	Bimolecular rate constant for degradation of the complex of β–catenin and Lef1		0.442
$a_{\beta\|L}$	Bimolecular rate constant for binding of β–catenin to Lef1		5.59
v_{mAxin2}	Basal rate of transcription of the Axin2 gene		0.09
$V_{MsmAxin}$	Maximum rate of transcription of Axin2 gene induced by the complex of β–catenin and Lef1 in nucleus		2.46
$K_{smAxin2}$	Threshold constant for induction by the complex of β–catenin and Lef1 in nucleus of Axin2 transcription		0.5
$h3$	Hill coefficient of transcription of Axin2 induced by the complex of β–catenin and Lef1		2
v_{mDll1}	Basal rate of transcription of the Dll1 gene		1
$V_{MsmDll1}$	Maximum rate of transcription of Dll1 gene induced by the complex of β–catenin and Lef1 in nucleus		1.12
K_{smDll1}	Threshold constant for induction by the complex of β–catenin and Lef1 in nucleus of Dll1 transcription		0.24
$h4$	Hill coefficient of transcription of Dll1 induced by the complex of β–catenin and Lef1		2
V_{MdA}	Maximum rate of degradation of A	Dll1	0.9
		$mAxin2_c$	1.2

		$mDLL1_c$	2
K_{dA}	Michaelis constant for degradation of A	Dll1	0.336
		$mAxin2_c$	0.48
		$mDLL1_c$	1

First order rate constants are in units of min^{-1} and second order rate constants are expressed in $nM^{-1}min^{-1}$. Michaelis constants and total concentration of species are in nM. Hill coefficients are no units.

The parameter values noted by green font color come from the researching results of Albert Goldbeter at el. [1]. The parameter values noted by black font color are derived from the parameter learning algorithm.

Table S4. Initial values of the single Wnt pathway model

**Name**	**Description**	**Value (nM)**
Dsh_{act}	The active Dsh	0.426
Dsh_{inact}	The inactive Dsh	0.158
Axin2	The Axin2 protein	0.023
D|A	The complex of Dsh and Axin2	2.746
GSK3	The GSK3 protein	1.942
G|A	The complex of GSK3 and Axin2	2.083
$\beta\text{-cat}_p$	The phosphorylated β-catenin protein	0.005
β-cat	The unphosphorylated β-catenin protein	0.021
Dll1	The Dll1 protein	0.163
$mAxin2_c$	The Axin2 mRNA in the cytoplasm	0.33
$mDll1_c$	The Dll1 mRNA in the cytoplasm	2.961
$\beta\text{-cat}_n$	The β-catenin protein in the nucleus	0.008
$Lef1_n$	The Lef1 protein in the nucleus	2.473
β|L	The complex of β-catenin and Lef1	0.227
$mAxin2_n$	The Axin2 mRNA in the nucleus	0.251
$mDll1_n$	The Dll1 mRNA in the nucleus	0.882

Table S5. Parameters of the combined model except these contained in the single model

**Name**	**Description**		**Value**
$d_{N\|D}$	Bimolecular rate constant for degradation of the complex of NICD and Dsh		0.5
$a_{N\|D}$	Bimolecular rate constant for binding of NICD to Dsh		9
k_{mNkd1}	Apparent first-order rate constant for synthesis of Nkd1 protein		0.117
$d_{K\|D}$	Bimolecular rate constant for degradation of the complex of Nkd1 and Dsh		0.5
$a_{K\|D}$	Bimolecular rate constant for binding of Nkd1 to Dsh		2.25
EX_{mNkd1n}	Apparent first-order rate constant for Nkd1 mRNA exit from the nucleus		10
v_{mNkd1}	Basal rate of transcription of the Nkd1 gene		0.05
$V_{MsmNkd1}$	Maximum rate of transcription of Nkd1 gene induced by the complex of β–catenin and Lef1 in nucleus		2.1
K_{smNkd1}	Threshold constant for induction by the complex of β–catenin and Lef1 in nucleus of Nkd1 transcription		0.4
$h5$	Hill coefficient of transcription of Nkd1 induced by the complex of β–catenin and Lef1		2
$K_{Hes7ImNkd1n}$	Constant of inhibition by Hes7 of Nkd1 mRNA synthesis		0.2
$h_{Hes7ImNkd1n}$	Hill coefficient of inhibition by Hes7 of Nkd1 mRNA synthesis		2
V_{MdA}	Maximum rate of degradation of A	Dll1	0.9
		Nkd1	0.438
		$mNkd1_c$	1
K_{dA}	Michaelis constant for degradation of A	Dll1	0.336

		Nkd1	5
		$mNkd1_c$	0.74

First order rate constants are in units of min^{-1} and second order rate constants are expressed in $nM^{-1}min^{-1}$. Michaelis constants are in nM. Hill coefficients are no units. The parameter values are derived from the parameter learning algorithm.

Table S6. Initial values of the combined model

**Name**	**Description**	**Value (nM)**
Dll1	The Dll1 protein	1.68
Notch	The Notch protein	0.12
$NICD_c$	The NICD in the cytoplasm	0.001
Lfng	The Lfng protein	1.95
$Hes7_c$	The Hes7 protein in the cytoplasm	9.69
$mLfng_c$	The Lfng mRNA in the cytoplasm	0.04
$mHes7_c$	The Hes7 mRNA in the cytoplasm	0.04
RBP-j	The RBP-j protein	0.32
$NICD_n$	The NICD in the nucleus	0.015
N|R	The complex of NICD and RBP-j	0.03
$Hes7_n$	The Hes7 protein in the nucleus	1.2
$mLfng_n$	The Lfng mRNA in the nucleus	0.07
$mHes7_n$	The Hes7 mRNA in the nucleus	0.01
Dsh_{act}	The active Dsh	0.52
Dsh_{inact}	The inactive Dsh	0.18
Axin2	The Axin2 protein	0.018
D|A	The complex of Dsh and Axin2	1.96
GSK3	The GSK3 protein	2.82
G|A	The complex of GSK3 and Axin2	1.2
$\beta\text{-cat}_p$	The phosphorylated β-catenin protein	0.005
β-cat	The unphosphorylated β-catenin protein	0.04

$mAxin2_c$	The Axin2 mRNA in the cytoplasm	3.6
$mDll1_c$	The Dll1 mRNA in the cytoplasm	7.57
$\beta\text{-cat}_n$	The β-catenin protein in the nucleus	0.017
$Lef1_n$	The Lef1 protein in the nucleus	2.26
β|L	The complex of β-catenin and Lef1	0.47
$mAxin2_n$	The Axin2 mRNA in the nucleus	0.62
$mDll1_n$	The Dll1 mRNA in the nucleus	1.09
Nkd1	The Nkd1 protein	0.27
N|D	The complex of NICD and Dsh	0.02
K|D	The complex of Nkd1 and Dsh	0.65
$mNkd1_c$	The Nkd1 mRNA in the cytoplasm	0.07
$mNkd1_n$	The Nkd1 mRNA in the nucleus	0.013

1. Goldbeter A, Pourquie O: **Modeling the segmentation clock as a network of coupled oscillations in the Notch, Wnt and FGF signaling pathways**. *Journal of Theoretical Biology* 2008, **252**:574-585.
