# Supplementary material for: Mathematical models for the Notch and Wnt signaling pathways and the crosstalk between them during somitogenesis
Source: Theor Biol Med Model. 2013 Apr 20;10:27. doi: 10.1186/1742-4682-10-27 (PMC3648501; doi:10.1186/1742-4682-10-27)
Supplement: Additional file 2 — The results of parameter sensitivity analysis.Figures S1 to S12 present the results of the parameter sensitivity analysis of the three models and Tables S7 to S9 contain the parameters to which the models are significantly sensitive. [file 1742-4682-10-27-S2.pdf]

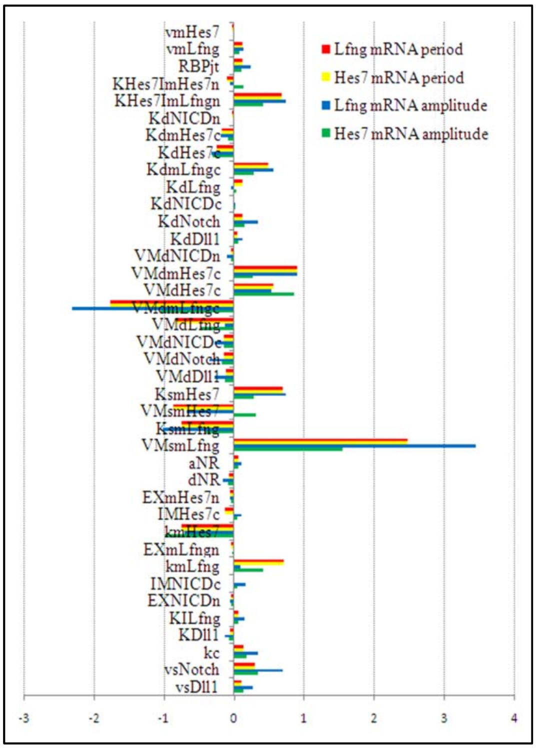

Figure S1. The parameter sensitivity of the single Notch pathway model under the perturbation level 1%.

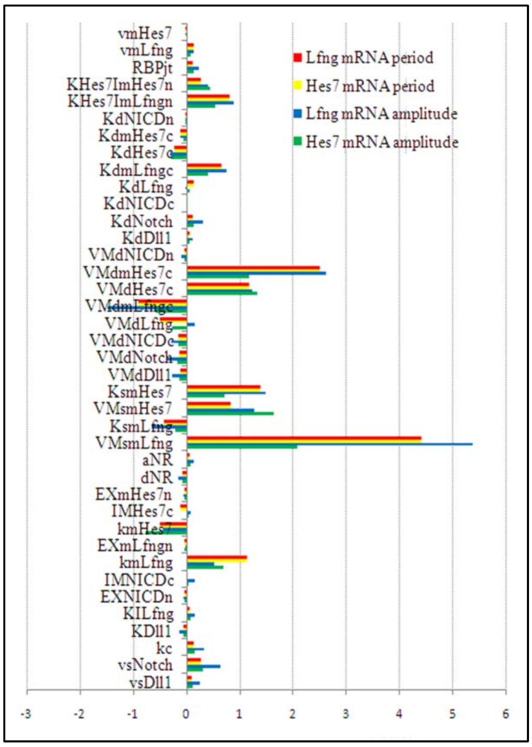

Figure S2. The parameter sensitivity of the single Notch pathway model under the perturbation level 10%.

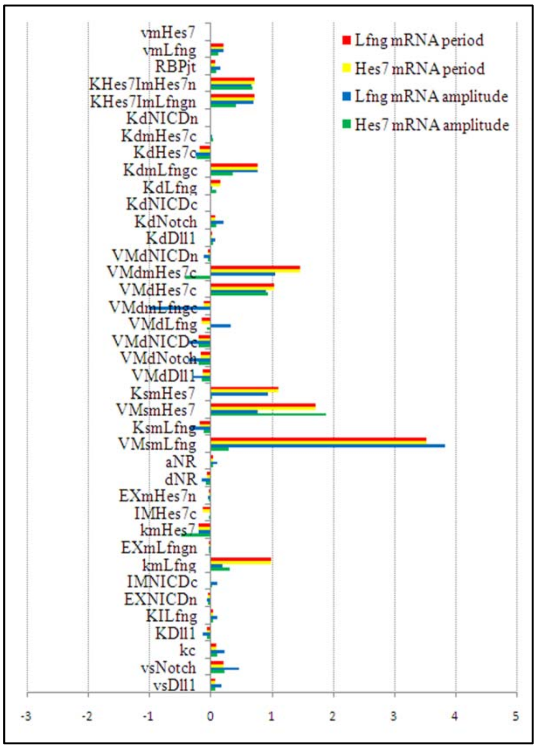

Figure S3. The parameter sensitivity of the single Notch pathway model under the perturbation level 50%.

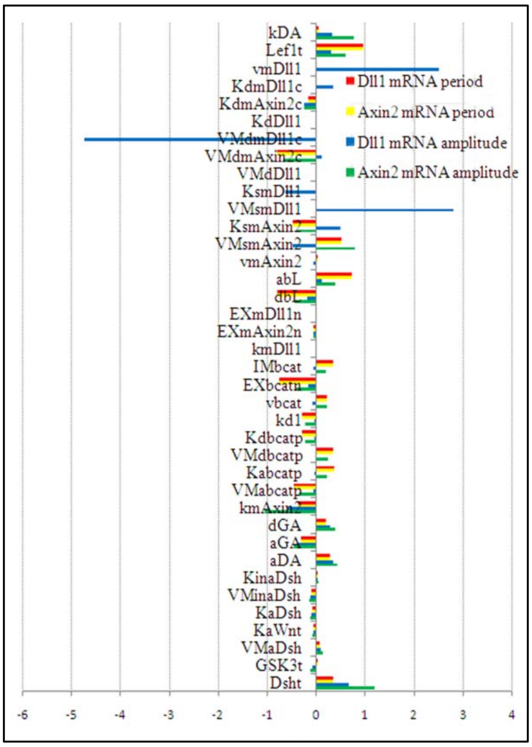

Figure S4. The parameter sensitivity of the single Wnt pathway model under the perturbation level 1%.

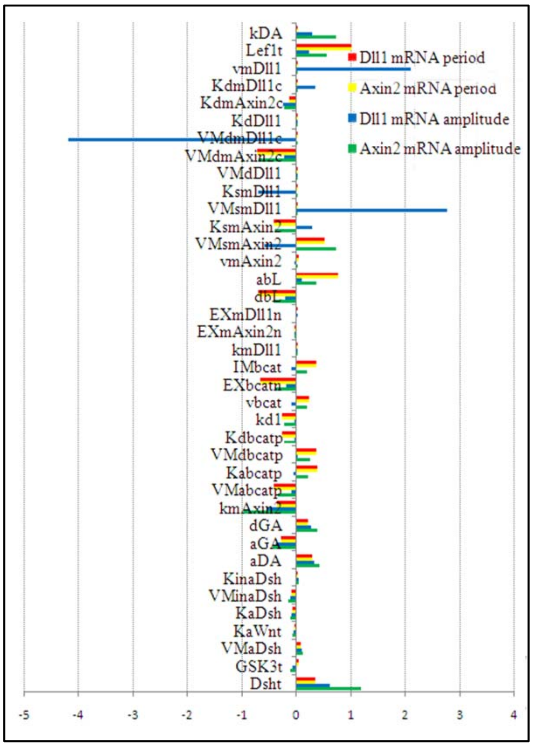

Figure S5. The parameter sensitivity of the single Wnt pathway model under the perturbation level 10%.

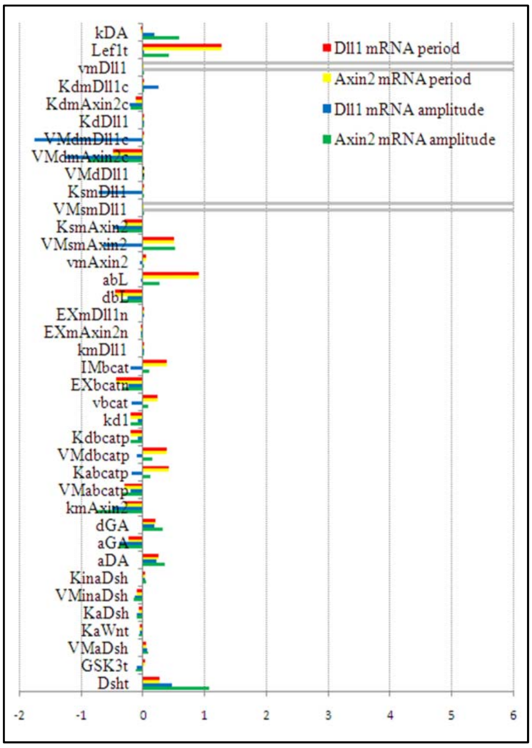

Figure S6. The parameter sensitivity of the single Wnt pathway model under the perturbation level 50%.

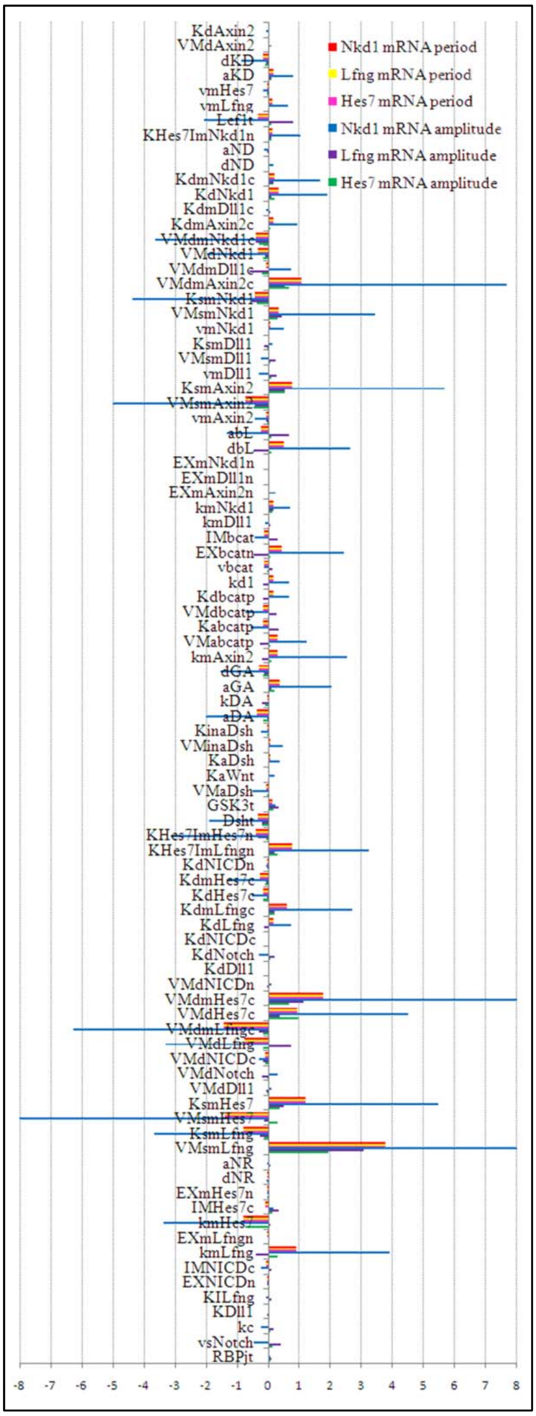

Figure S7. The parameter sensitivity of the combined model taking the genes oscillating synchronously with Hes7 as output under the perturbation level 1%.

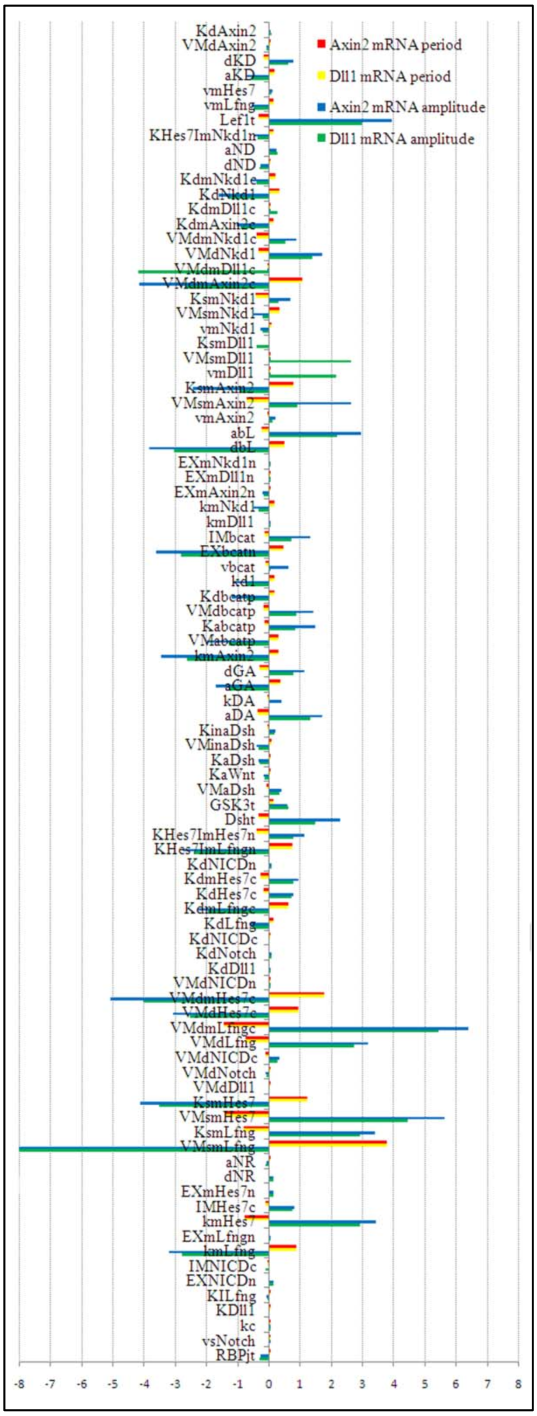

Figure S8. The parameter sensitivity of the combined model taking the genes oscillating synchronously with Axin2 as output under the perturbation level 1%.

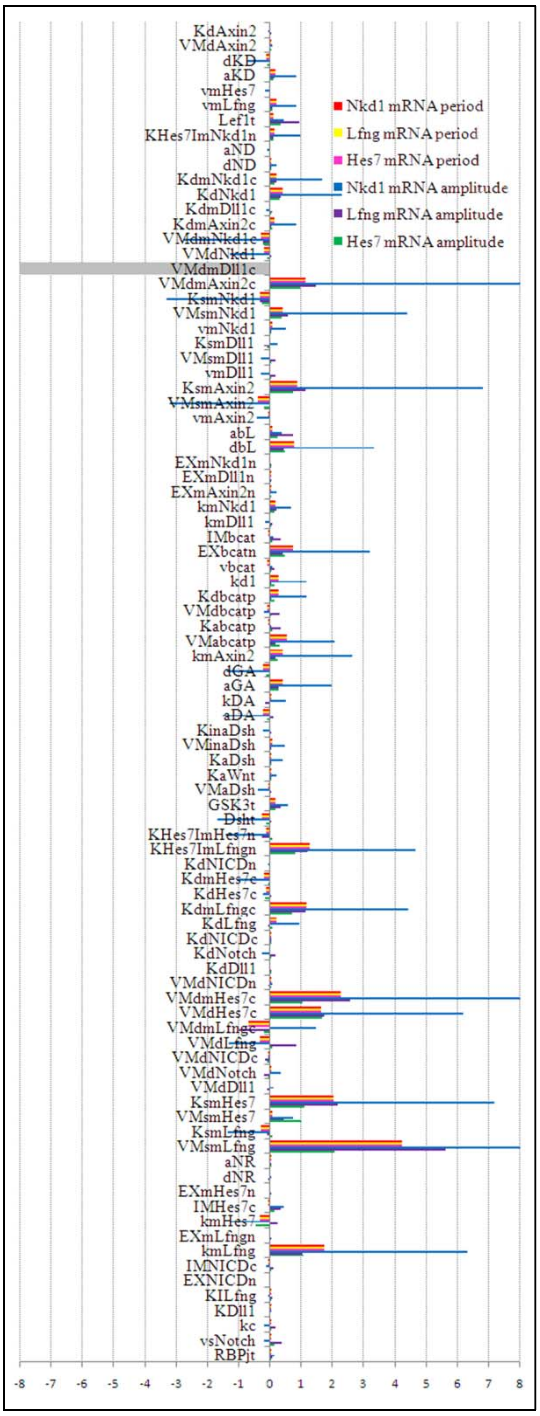

Figure S9. The parameter sensitivity of the combined model taking the genes oscillating synchronously with Hes7 as output under the perturbation level 10%.

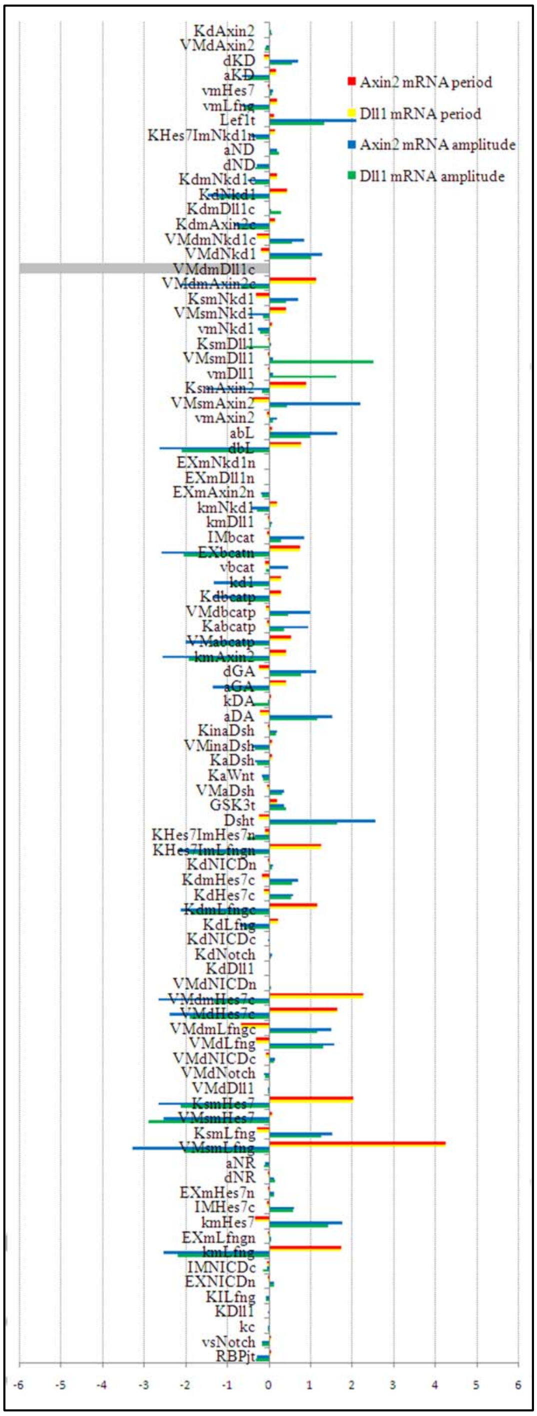

Figure S10. The parameter sensitivity of the combined model taking the genes oscillating synchronously with Axin2 as output under the perturbation level 10%.

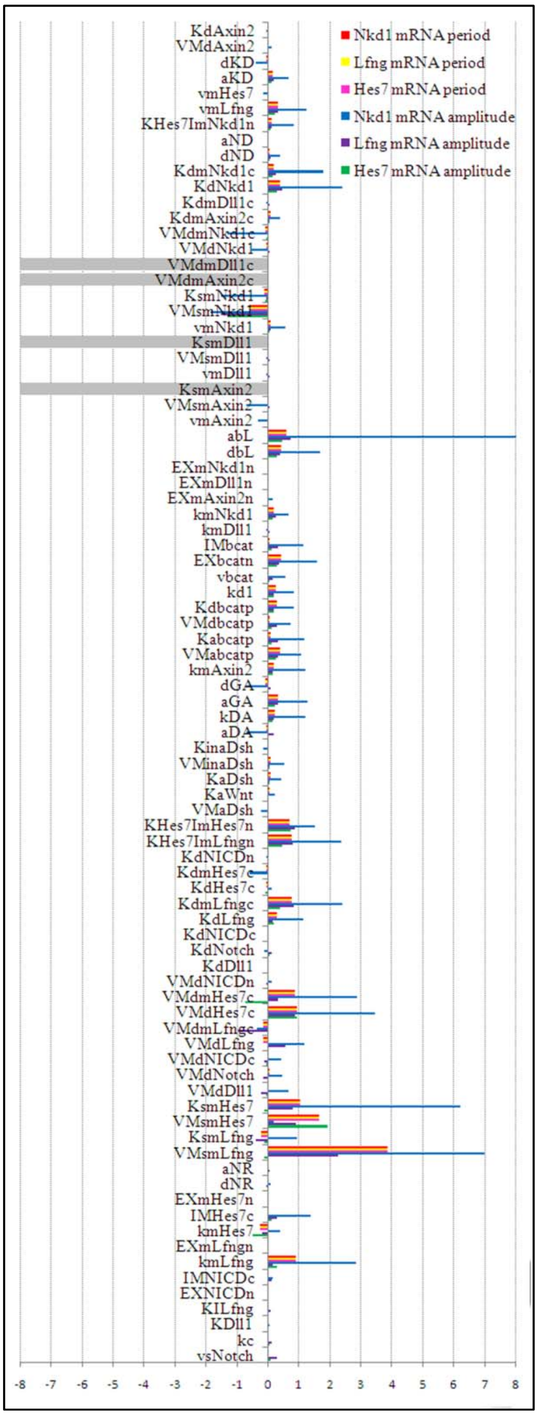

Figure S11. The parameter sensitivity of the combined model taking the genes oscillating synchronously with Hes7 as output under the perturbation level 50%.

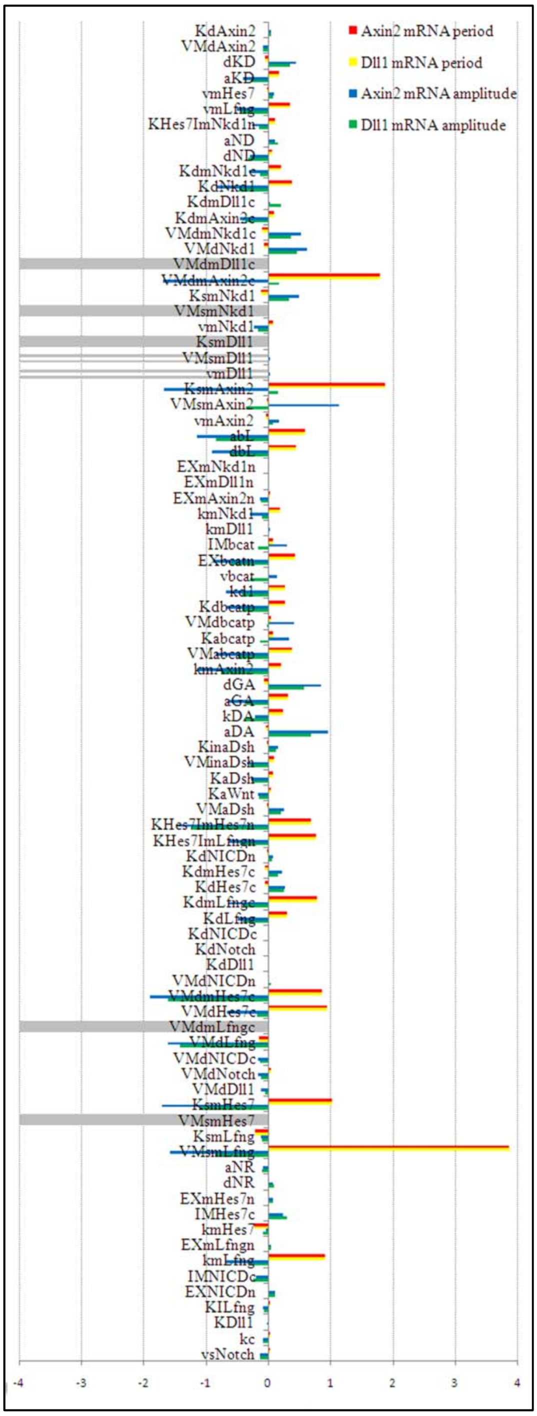

Figure S12. The parameter sensitivity of the combined model taking the genes oscillating synchronously with Axin2 as output under the perturbation level 50%.

Table S7. The parameters to which the oscillating periods or amplitudes of Notch target genes are significantly sensitive in different perturbation levels in the single Notch pathway model.

gene	Sensitivity type	Perturbation intensity		
		1%	10%	50%
**Lfng**	**period**	VMsmLfng(2.48)<br>VMdmLfngc(-1.76)	VMsmLfng(4.4)<br>VMdmHes7c(2.5)<br>KsmHes7(1.38)<br>VMdHes7c(1.16)<br>kmLfng(1.13)	VMsmLfng(3.52)<br>VMsmHes7(1.72)<br>VMdmHes7c(1.47)<br>KsmHes7(1.11)<br>VMdHes7c(1.04)
	**amplitude**	VMsmLfng(3.45)<br>VMdmLfngc(-2.31)<br>KsmLfng(-1.01)	VMsmLfng(5.36)<br>VMdmHes7c(2.62)<br>VMdmLfngc(-1.49)<br>KsmHes7(1.48)<br>VMsmHes7(1.27)<br>VMdHes7c(1.24)	VMsmLfng(3.83)<br>VMdmHes7c(1.07)<br>VMdmLfngc(-1)
**Hes7**	**period**	VMsmLfng(2.48)<br>VMdLfngc(-1.76)	VMsmLfng(2.08)<br>VMdmHes7c(2.5)<br>VMdHes7c(1.16)<br>KsmHes7(1.38)<br>kmLfng(1.13)	VMsmLfng(3.52)<br>VMsmHes7(1.72)<br>VMdmHes7c(1.47)<br>KsmHes7(1.11)<br>VMdHes7c(1.04)
	**amplitude**	VMsmLfng(1.55)<br>VMdmLfngc(-1.04)	VMsmLfng(4.4)<br>VMsmHes7(1.64)<br>VMdHes7c(1.33)<br>VMdmHes7c(1.17)	VMsmHes7(1.89)

Table S8. The parameters to which the oscillating periods or amplitudes of Wnt target genes are significantly sensitive in different perturbation levels in the single Wnt pathway model.

gene	Sensitivity type	Perturbation intensity		
		1%	10%	50%
**Dll1**	**period**		Lef1t(1.02)	Lef1t(1.27)
	**amplitude**	VMdmDll1c(-4.74)<br>VMsDll1(2.81)<br>vmDll1(2.52)	VMdmDll1c(-4.17)<br>VMsDll1(2.77)<br>vmDll1(2.1)	VMdmDll1c(-1.75)<br>VMdmAxin2c(-1.27)
**Axin2**	**period**		Lef1t(1.02)	Lef1t(1.27)
	**amplitude**	Dsht(1.21)<br>kmAxin2(-1.04)	Dsht(1.18)	Dsht(1.07)

Table S9. The parameters to which the oscillating periods or amplitudes of target genes are significantly sensitive in different perturbation levels in the combined model.

gene	Sensitivity type	Perturbation intensity		
		**1%**	**10%**	**50%**
**Lfng**	**period**	VMsmLfng(3.78)<br>VMdmHes7c(1.76)<br>VMdmLfngc(-1.45)<br>VMsmHes7(-1.41)<br>KsmHes7(1.22)<br>VMdmAxin2c(1.05)	VMsmLfng(4.24)<br>VMdmHes7c(2.28)<br>KsmHes7c(2.03)<br>kmLfng(1.72)<br>VMdHes7c(1.64)<br>KHes7ImLfngn(1.26)<br>KdmLfngc(1.16)<br>VMdmAxin2c(1.14)	VMsmLfng(3.88)<br>VMsmHes7(1.64)<br>KsmHes7(1.02)
	**amplitude**	VMsmLfng(3.07)<br>VMdmDll1c(-1.84)<br>VMdLfng(1.28)<br>Lef1t(1.27)<br>kmLfng(-1.12)<br>VMsmHes7(1.04)	VMsmLfng(5.61)<br>VMdmHes7c(2.56)<br>KsmHes7(2.19)<br>VMdHes7c(1.74)<br>VMdmAxin2c(1.48)<br>KHes7ImLfngn(1.2)<br>KsmAxin2(1.16)<br>KdmLfngc(1.13)<br>kmLfng(1.05)	VMsmLfng(2.27)<br>VMsmNkd1(-1.52)
**Hes7**	**period**	VMsmLfng(3.78)<br>VMdmHes7c(1.76)<br>VMdmLfngc(-1.45)<br>VMsmHes7(-1.41)<br>KsmHes7(1.22)<br>VMdmAxin2c(1.05)	VMsmLfng(4.24)<br>VMdmHes7c(2.28)<br>KsmHes7c(2.03)<br>kmLfng(1.72)<br>VMdHes7c(1.64)<br>KHes7ImLfngn(1.26)<br>KdmLfngc(1.16)<br>VMdmAxin2c(1.14)	VMsmLfng(3.88)<br>VMsmHes7(1.64)<br>KsmHes7(1.02)
	**amplitude**	VMsmLfng(1.94)<br>VMdmHes7c(1.15)	VMsmLfng(2.09)<br>VMdHes7c(1.68)<br>KsmHes7(1.13)<br>kmLfng(1.09)<br>VMdmHes7c(1.03)	VMsmHes7(1.9)<br>VMsmNkd1(-1.31)
**Nkd1**	**period**	VMsmLfng(3.78)<br>VMdmHes7c(1.76)<br>VMdmLfngc(-1.45)<br>VMsmHes7(-1.41)<br>KsmHes7(1.22)<br>VMdmAxin2c(1.05)	VMsmLfng(4.24)<br>VMdmHes7c(2.28)<br>KsmHes7c(2.03)<br>kmLfng(1.72)<br>VMdHes7c(1.64)<br>KHes7ImLfngn(1.26)<br>KdmLfngc(1.16)	VMsmLfng(3.88)<br>VMsmHes7(1.64)<br>KsmHes7(1.02)

			VMdmAxin2c(1.14)	
	**amplitude**	VMsmLfng(15.56)<br>VMdmHes7c(9.41)<br>VMsmHes7(-8.51)<br>VMdmAxin2c(7.68)<br>VMdmLfngc(-6.29)<br>KsmAxin2(5.67)<br>KsmHes7(5.48)<br>VMsmAxin2(-5.01)<br>VMdHes7c(4.52)<br>KsmNkd1(-4.36)<br>KsmLfng(-3.67)<br>VMdmNkd1c(-3.66)<br>VMsmNkd1(3.42)<br>kmHes7(-3.39)<br>VMdLfng(-3.32)<br>KHes7ImLfngn(3.23)<br>KHes7ImHes7n(-3.16)<br>kmLfng(3.01)<br>KdmLfngc(2.7)<br>dbL(2.65)<br>kmAxin2(2.55)<br>EXbcatn(2.43)<br>aGA(2.04)<br>aDA(-2)<br>VMdNkd1(-1.98)<br>KdNkd1(1.91)<br>KdmNkd1c(1.65)<br>dGA(-1.55)<br>abL(-1.33)<br>KdmHes7c(-1.32)<br>VMabcatp(1.24))<br>KdmNkd1c(1.09)<br>KHes7ImNkd1n(1.02)	VMsmLfng(12.13)<br>VMdmHes7c(10.79)<br>VMdmAxin2c(8.8)<br>KsmHes7(7.18)<br>kmLfng(6.32)<br>VMdHes7c(6.19)<br>KHes7ImLfngn(4.66)<br>KdmLfngc(4.42)<br>VMsmNkd1(4.4)<br>dbL(3.34)<br>KsmNkd1(-3.29)<br>EXbcatn(3.2)<br>VMsmAxin2(-3.19)<br>VMdmNkd1c(-2.78)<br>kmAxin2(2.63)<br>KdNkd1(2.31)<br>VMabcatp(2.09)<br>aGA(1.99)<br>KdmNkd1c(1.68)<br>aDA(-1.52)<br>VMdmLfngc(1.48)<br>KHes7ImHes7n(-1.41)<br>dGA(-1.36)<br>KsmLfng(-1.36)<br>VMdLfng(-1.3)<br>kmHes7(-1.29)<br>VMdNkd1(-1.27)<br>Kdbcatp(1.18)<br>kd1(1.17)<br>KsmAxin2(1.16)	abL(10.16)<br>VMsmLfng(7.01)<br>KsmHes7(6.21)<br>VMdHes7c(3.44)<br>VMdmHes7c(2.87)<br>kmLfng(2.83)<br>KdNkd1(2.41)<br>KdmLfngc(2.38)<br>KHes7ImLfngn(2.38)<br>VMsmNkd1(-1.85)<br>KdmNkd1c(1.78)<br>dbL(1.68)<br>EXbcatn(1.58)<br>KHes7ImHes7n(1.51)<br>KsmNkd1(-1.51)<br>VMdmNkd1c(-1.31)<br>aGA(1.29)<br>vmLfng(1.23)<br>kDA(1.22)<br>kmAxin2(1.2)<br>VMdLfng(1.18)<br>Kabcatp(1.18)<br>KdLfng(1.14)<br>IMbcat(1.13)<br>VMabcatp(1.07)
**Dll1**	**period**	VMsmLfng(3.78)<br>VMdmHes7c(1.76)<br>VMdmLfngc(-1.45)<br>VMsmHes7(-1.41)<br>KsmHes7(1.22)<br>VMdmAxin2c(1.05)	VMsmLfng(4.24)<br>VMdmHes7c(2.28)<br>KsmHes7c(2.03)<br>kmLfng(1.72)<br>VMdHes7c(1.64)<br>KHes7ImLfngn(1.26)<br>KdmLfngc(1.16)<br>VMdmAxin2c(1.14)	VMsmLfng(3.88)<br>VMsmHes7(1.64)<br>KsmHes7(1.02)
	**amplitude**	VMsmLfng(-9.58)<br>VMdmLfngc(5.43)	VMsmHes7(-2.9)<br>VMsDll1(2.5)	VMdmHes7c(-1.61)<br>KHes7Imhes7n(-1.24)

		VMsmHes7(4.45)<br>dbL(-3.06)<br>abL(2.18)<br>KsmHes7(-3.52)<br>EXbcatn(-2.83)<br>VMdmDll1c(-4.2)<br>VMdmAxin2c(-2.68)<br>VMdmHes7c(-4.01)<br>KsmLfng(2.9)<br>kmHes7(2.92)<br>VMdLfng(2.72)<br>kmAxin2(-2.64)<br>VMsmDll1(2.62)<br>VMdHes7c(-2.54)<br>kmLfng(-2.79)<br>VMabcatp(-1.29)<br>KHes7ImLfngn(-2.41)<br>vmDll1(2.13)<br>aDA(1.32)<br>KdmLfngc(-1.97)<br>aGA(-1.3)<br>VMdNkd1(1.4)<br>KdNkd1(-1.32)	kmLfng(-2.2)<br>KsmHes7(-2.12)<br>dbL(-2.11)<br>EXbcatn(-2.05)<br>VMsmLfng(-2.03)<br>VMdmLfngc(1.15)<br>KHes7ImLfngn(-1.96)<br>kmAxin2(-1.94)<br>VMdHes7c(-1.91)<br>KdmLfngc(-1.89)<br>vmDll1(1.62)<br>kmHes7(1.42)<br>VMabcatp(-1.4)<br>VMdmHes7c(-1.33)<br>VMdLfng(1.3)<br>KsmLfng(1.27)<br>KdNkd1(-1.21)<br>aDA(1.15)<br>VMdNkd1(1.02)	VMdLfng(-1.42)<br>KsmHes7(-1.09)
**Axin2**	**period**	VMsmLfng(3.78)<br>VMdmHes7c(1.76)<br>VMdmLfngc(-1.45)<br>VMsmHes7(-1.41)<br>KsmHes7(1.22)<br>VMdmAxin2c(1.05)	VMsmLfng(4.24)<br>VMdmHes7c(2.28)<br>KsmHes7c(2.03)<br>kmLfng(1.72)<br>VMdHes7c(1.64)<br>KHes7ImLfngn(1.26)<br>KdmLfngc(1.16)<br>VMdmAxin2c(1.14)	VMsmLfng(3.88)<br>VMsmHes7(1.64)<br>KsmHes7(1.02)
	**amplitude**	VMsmLfng(-10.8)<br>VMdmLfngc(6.38)<br>VMsmHes7(5.61)<br>abL(2.94)<br>dbL(-3.85)<br>VMdmAxin2c(-4.16)<br>EXbcatn(-3.61)<br>VMdmHes7c(-5.08)<br>KsmHes7(-4.13)<br>KsmLfng(3.38)<br>kmHes7(3.42)<br>kmAxin2(-3.46)<br>VMsmAxin2(2.63)	VMsmLfng(-3.28)<br>VMdmHes7c(-2.65)<br>KsmHes7(-2.65)<br>dbL(-2.63)<br>EXbcatn(-2.57)<br>kmAxin2(-2.56)<br>VMsmHes7(-2.53)<br>kmLfng(-2.53)<br>VMdHes7c(-2.39)<br>KHes7ImLfngn(-2.2)<br>VMsmAxin2(2.19)<br>KdmLfngc(-2.13)<br>VMdmAxin2c(-2.13)	VMsmLfng(-1.59)<br>abL(-1.14)<br>VMdmHes7c(-1.9)<br>KHes7ImHes7n(-1.48)<br>VMdLfng(-1.62)<br>VMsmAxin2(1.14)<br>KsmHes7(-1.71)<br>kmAxin2(-1.14)

		VMabcatp(-2)<br>VMdLfng(3.17)<br>KsmAxin2(-2.43)<br>Kabcatp(1.47)<br>VMdHes7c(-3.07)<br>IMbcat(1.32)<br>kmLfng(-3.21)<br>VMdbcatp(1.43)<br>aDA(1.71)<br>KHes7ImLfngn(-2.76)<br>aGA(-1.7)<br>KdmLfngc(-2.26)<br>Kdbcatp(-1.18)<br>kd1(-1.18)<br>VMdNkd1(2.15)<br>dGA(1.14)<br>KdNkd1(-1.59)<br>KHes7IHes7n(1.13)<br>KdmAxin2c(-1.04)	VMabcatp(-2)<br>kmHes7(1.75)<br>abL(1.64)<br>VMdLfng(1.58)<br>KsmAxin2(-1.53)<br>KsmLfng(1.52)<br>aDA(1.51)<br>VMdmLfngc(1.49)<br>KdNkd1(-1.48)<br>aGA(-1.35)<br>Kdbcatp(-1.34)<br>kd1(-1.33)<br>VMdNkd1(1.28)<br>aGA(-1.14)	
