# Supplementary material for: Mathematical models for the Notch and Wnt signaling pathways and the crosstalk between them during somitogenesis
Source: Theor Biol Med Model. 2013 Apr 20;10:27. doi: 10.1186/1742-4682-10-27 (PMC3648501; doi:10.1186/1742-4682-10-27)
Supplement: Additional file 4 — The pseudocode of the parameter learning algorithm. This file contains the pseudocode of the parameter learning algorithm proposed in this paper, which is described by a process design language. [file 1742-4682-10-27-S4.pdf]

The algorithm pseudocode of parameter learning algorithm

1. Initialize the control parameters of the algorithm: *popCount, maxIter, Nc,* δ, θ, $c1$, $c2$, w, v_{max} and v_{min}.

2. Random initialize the population *P* of the PSO.

3. Calculate the fitness value of each particle and save the values to *f*.

   If it is the first iteration then *T*=max(*f*)

4. Update the best value of each particle *fpBest* and the best particle *pBest*.

   For each particle:

   (1) if current value *f* < *fpBest* then *fpBest*← *f* , *pBest*← *p*;

   (2) else if *f* > *pBest* then:

      a. *ΔS* ← *f* - *fpBest*;

      b. *s* ← exp(-*ΔS*/*T*);

      c. *r* ← random value greater than 0 and less than 1;

      d. if *r* < *s* then *fpBest*←*f*, *pBest*← *p*;

5. Update the global best value *fgBest* and the best particle *gBest*.

   (1) If minimum value of all current values min(*fpBest*) < *fgBest* then *fgBest* ← min(*fpBest*), *gBest* ← *pBest* who has the minimum value;

   (2) Else if min(*fpBest*) > *fgBest* then:

      a. *ΔS* ← min(*fpBest*) - *fgBest*;

      b. *s* ← exp(-*ΔS*/*T*);

      c. *r* ← random value greater than 0 and less than 1;

      d. if *r* < *s* then *fgBest* ← min(*fpBest*), *gBest* ← *pBest* who has the minimum

value;

6. Random update the particles that are the same as the one that has the best value.

   For each particle *p*:

   If *p=gBest* then:

   a. *vv* ← random value between v_{max} and v_{min};

   b. *p* ← *p+vv*;

7. Update *P* using the *pBest* and *gBest*.

   (1) *r1* ← random value greater than 0 and less than 1;

   (2) *r2* ← random value greater than 0 and less than 1;

   (3) $v \leftarrow v*w+c1*r1*(pBest-P)+c2*r2*(gBest-P)$;

   (4) if $v > v_{max}$ then $v \leftarrow v_{max}$; if $v < v_{min}$ then $v \leftarrow v_{min}$;

   (5) $P \leftarrow P+v$;

8. Check the evolution of *fpBest*:

   If the current iteration counts $iter >= Nc$ and $|fpBest(i)-fpBest(i-1)| < \delta$ for each $i=iter, iter-1, \ldots, iter-Nc+1$ then:

   a. *vv*← random value between v_{max} and v_{min};

   b. Random select some particles *pp*, *pp* ← *pp+vv*;

9. Update the temperature *T*.

   If the iteration passes the half of remaining iteration counts when T is updated last time then $T \leftarrow T/2$;

10. If $fgBest < \theta$ then go to 12.

11. If $iter > maxIter$ then GOTO step 12, else GOTO step 3.

12. Reinitialize the control parameters of the algorithm: *maxIter*, *T*, *updateNeiCount*, neiCount, v_{max} and v_{min}.
13. Initialize the population *P* of the PSO using the result of global search, *gBest*.
14. Calculate the fitness value of each particle.
15. Update the best value of each particle *fpBest* and the best particle *pBest*.

    For each particle:

    (1) if current value $f < fpBest$ then $fpBest \leftarrow f$, $pBest \leftarrow p$;

    (2) else if $f > pBest$ then:

    a. $\Delta S \leftarrow f - fpBest$;

    b. $s \leftarrow \exp(-\Delta S/T)$;

    c. $r \leftarrow$ random value greater than 0 and less than 1;

    d. if $r < s$ then $fpBest \leftarrow f$, $pBest \leftarrow p$;

16. Update the local best value of each neighbour *flBest* and the best particle *lBest* of each neighbour.

    Create neighbour for each particle and for each neighbour:

    (1) If minimum value of current values in this neighbour $\min(fpBest) < flBest$ then $flBest \leftarrow \min(fpBest)$, $lBest \leftarrow pBest$ that has minimum value in the neighbour;

    (2) Else if $\min(fpBest) > flBest$ then:

    a. $\Delta S \leftarrow \min(fpBest) - flBest$;

    b. $s \leftarrow \exp(-\Delta S/T)$;

    c. $r \leftarrow$ random value greater than 0 and less than 1;

d. if $r < s$ then *flBest* ← min(*fpBest*), *lBest* ← *pBest* that has minimum value in the neighbour;

17. Output the *fgBest* and *gBest*.

(1) *fgBest* ← minimum value of *flBest*;

(2) *gBest* ← *p* that has the best value *fgBest*;

18. Update *P* using the *pBest* and *lBest*.

(1) *r1* ← random value greater than 0 and less than 1;

(2) *r2* ← random value greater than 0 and less than 1;

(3) *v* ← *v*w+c1*r1**(*pBest-P*)+*c2*r2**(*lBest-P*);

(4) if $v > v_{max}$ then $v \leftarrow v_{max}$; if $v < v_{min}$ then $v \leftarrow v_{min}$;

(5) *p* ← *p+v*;

19. Check the evolution of *fpBest.*

If the current iteration counts *iter* >=*Nc* and |*fpBest*(*i*)-*fpBest*(*i-1*)| < δ for each *i=iter, iter-1, …, iter-Nc+1* then:

a. *vv* ← random value between v_{max} and v_{min};

b. Random select some particles *pp*, *pp* ← *pp+vv*;

20. Update the neighbour count *neiCount*.

If *iter* mod *updateNeiCount* = 0 then *neiCount* ← *neiCount*+1

21. Update the temperature *T*.

If the iteration passes the half of remaining iteration counts when *T* is updated last time then *T* ← *T/2*;

If *iter* > *maxIter or fgBest* <θ then output *fgBest* and *gBest*, the algorithm is over; else

GOTO step 14.
